# Membrane potential drives the exit from pluripotency and cell fate commitment via calcium and mTOR

**DOI:** 10.1038/s41467-022-34363-w

**Published:** 2022-11-05

**Authors:** Emily Sempou, Valentyna Kostiuk, Jie Zhu, M. Cecilia Guerra, Leonid Tyan, Woong Hwang, Elena Camacho-Aguilar, Michael J. Caplan, David Zenisek, Aryeh Warmflash, Nick D. L. Owens, Mustafa K. Khokha

**Affiliations:** 1 Pediatric Genomics Discovery Program, Departments of Pediatrics and Genetics, Yale University School of Medicine, 333 Cedar Street, New Haven, CT 06510 USA; 2 Department of Cellular and Molecular Physiology, Yale University School of Medicine, 333 Cedar Street, New Haven, CT 06510 USA; 3 Departments of Biosciences and Bioengineering Rice University, 345 Anderson Biological Labs, Houston, TX 77005 USA; 4 Department of Clinical and Biomedical Sciences, University of Exeter, Barrack Road, Exeter, EX2 5DW UK

**Keywords:** Pluripotency, Embryology, Mesoderm, Ion channel signalling

## Abstract

Transitioning from pluripotency to differentiated cell fates is fundamental to both embryonic development and adult tissue homeostasis. Improving our understanding of this transition would facilitate our ability to manipulate pluripotent cells into tissues for therapeutic use. Here, we show that membrane voltage (V_m_) regulates the exit from pluripotency and the onset of germ layer differentiation in the embryo, a process that affects both gastrulation and left-right patterning. By examining candidate genes of congenital heart disease and heterotaxy, we identify *KCNH6*, a member of the ether-a-go-go class of potassium channels that hyperpolarizes the V_m_ and thus limits the activation of voltage gated calcium channels, lowering intracellular calcium. In pluripotent embryonic cells, depletion of *kcnh6* leads to membrane depolarization, elevation of intracellular calcium levels, and the maintenance of a pluripotent state at the expense of differentiation into ectodermal and myogenic lineages. Using high-resolution temporal transcriptome analysis, we identify the gene regulatory networks downstream of membrane depolarization and calcium signaling and discover that inhibition of the mTOR pathway transitions the pluripotent cell to a differentiated fate. By manipulating V_m_ using a suite of tools, we establish a bioelectric pathway that regulates pluripotency in vertebrates, including human embryonic stem cells.

## Abstract (web summary)

The plasma membrane’s electrical potential is maintained by ion channels, though the impact of this potential on cell fate has not been clearly elucidated. Here they show that changes in membrane potential can affect calcium levels and mTOR in pluripotent stem cells, altering their transition from pluripotency to differentiation.

## Introduction

**Fig. 1:**
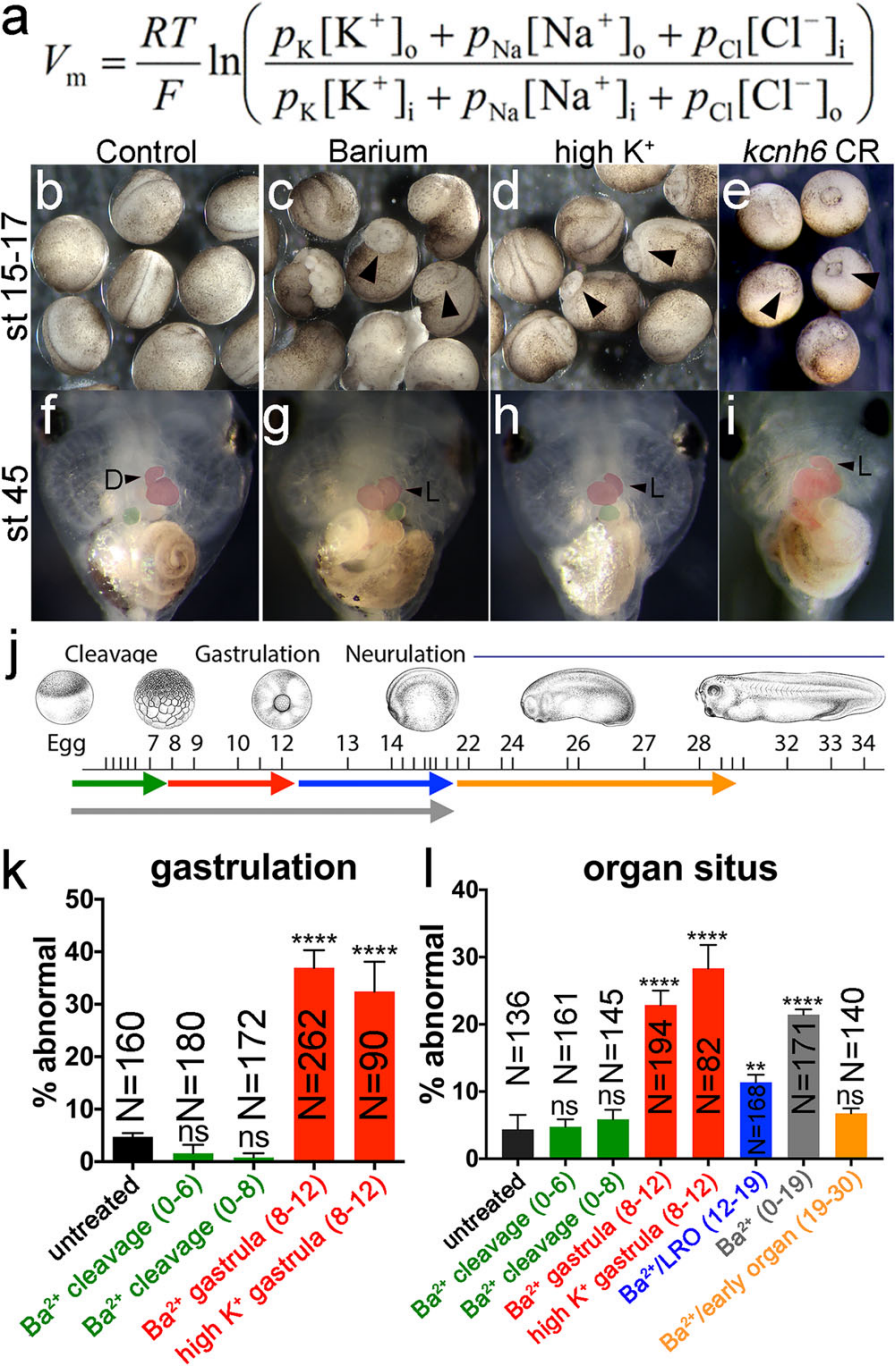
Membrane potential is important for gastrulation and LR patterning. **a** GHK equation for V_m_; R = gas constant, T = temperature, F = Faraday’s constant, *p* = permeability for each ion, [X]_o_ = ion concentration outside of cell, [X]_i_ = ion concentration inside the cell. **b**–**i** Effects of depolarizing treatments (barium chloride and high K^+^) and *kcnh6* depletion on gastrulation (**b**–**e**; stage 15–17 embryos; arrowheads indicate incomplete blastopore closure) and organ *situs* (**f**–**i**; stage 45 tadpoles; ventral views; arrowheads indicate normal (D) and inverse (L) heart looping). **j** Different stages of barium chloride application (color key in **j** for bar graphs **k** and **l**; green = cleavage stages (stages 0–6 or 0–8); red = gastrulation (stages 8–12); blue = LRO signaling (stages 12–19); orange = early organogenesis (stages 19–30); gray = cleavage through LRO signaling (stages 0–19). *Xenopus* illustrations © Natalya Zahn (2022) from Xenbase (www.xenbase.org RRID:SCR_003280). **k**, **l** Percentages of embryos with incomplete blastopore closure at stage 15 (**k**) and abnormal organ situs at stage 45 (**l**) after treatment with barium or high K^+^ at different stages (see **j** for color code); *p*-values are in **k**: (Ba^2+^ gastrula vs untreated) = 2.28e–015, (high K^+^ gastrula vs untreated) = 1.36e–006; in **l**: (Ba^2+^ gastrula vs untreated) = 5.85e–007, (high K^+^ gastrula vs untreated) = 2.08e–008, (Ba^2+^/LRO vs untreated) = 8.51e–003, and (Ba^2+^ 0–19 vs untreated) = 1.2e–006. All graphs depict mean ± SEM and report total embryo numbers (N) collected over 3 independent experiments for high K^+^, and over 2 independent experiments for the 47 h barium time course. Key for asterisks: *p ≤ 0.05, **p ≤ 0.01, ****p ≤ 0.0001, ns nonsignificant with *p* > 0.05. Source data are provided as a Source Data file.

Action potentials are fundamental to the function of excitable cells, including neurons, cardiomyocytes, and pancreatic cells. They are produced through tightly orchestrated changes in the membrane potential (V_m_). However, most animal cells, excitable or not, have a resting state V_m_ (resting membrane potential) that depends on (a) the permeability of the plasma membrane for each ion (*p* in the Goldman-Hodgkin-Katz (GHK) equation, Fig. 1a, indicating the number of active ion channels), and (b) the driving force for each ion across the plasma membrane, determined by its electrochemical gradient (e.g., [K]_i_ vs. [K]_o_ in GHK equation). Although molecules that influence membrane potential have established roles in excitable tissues, their functions in embryonic or adult “non-excitable” tissues are emerging. For example, ion channels and pumps appear to be crucial for the formation of the left-right (LR) body axis^1^. While the vertebrate body plan may appear symmetrical across the LR axis, some of our internal organs, including the heart and gut, require asymmetry across the LR axis for proper formation or function. Chemical inhibition or overexpression of ion channels or pumps disrupt the proper alignment of internal organs along the left-right axis and affect global LR patterning^2–7^. Notably, using voltage sensitive dyes, V_m_ appears to vary across the developing embryo suggesting it could play instructive roles^3,6,8^. There is now a growing field that has implicated V_m_ in various embryonic contexts including Drosophila wing patterning^8,9^, craniofacial morphogenesis^10,11^, and chondrogenesis^12,13^ as well as the differentiation of excitable tissues such as muscle cells and neurons^14^. A challenge in the field is connecting changes in V_m_ to voltage responsive effectors that lead to the gene expression changes that pattern the embryo.

In order to respond, voltage sensitive effector molecules depend on the magnitude in the change of V_m_. Quantitative V_m_ measurements in early embryos are rare but were performed in the 1960s and 1970s from 1-cell stage embryos through blastula stages in *Triturus* and *Xenopus* embryos^15,16^. The blastula embryo has completed a series of rapid cell divisions (cleavages), has established germ-layer cell fates (ectoderm, mesoderm, and endoderm), and is poised to begin gastrulation, the process by which cell movements transform the embryo to acquire the adult body plan. Notably, while the V_m_ at early cleavage stages is depolarized (=more positive V_m_) (V_m@2-cell_ = −19 ± 10 mV), it becomes progressively hyperpolarized (=more negative) towards blastula stages (−50 mV)^16^. The implications of this progressive V_m_ polarization during early development are unclear, as is a mechanism by which V_m_ could transduce a signal within embryonic cells or act complementary to signals transduced biochemically (i.e., ligand-receptor).

Here we show that the V_m_ established in the blastula is essential for LR patterning and the exit from pluripotency. Depolarization of V_m_ using a variety of approaches leads to loss of ectodermal and paraxial mesodermal cell fates due to a persistence of pluripotency in these tissues. Membrane depolarization leads to the opening of voltage-gated calcium channels elevating intracellular calcium and maintaining pluripotency. Using RNA-seq, we find that mTOR signaling is downstream of V_m_ and modulates the transition from pluripotency to differentiated cell fate. These results define an electrochemical signaling pathway that acts complementary to biochemical (ligand-receptor) signaling pathways that transition pluripotent embryonic stem cells to differentiated cell fates.

## Results

### V_m_ of the blastula regulates LR patterning and gastrulation

**Fig. 2:**
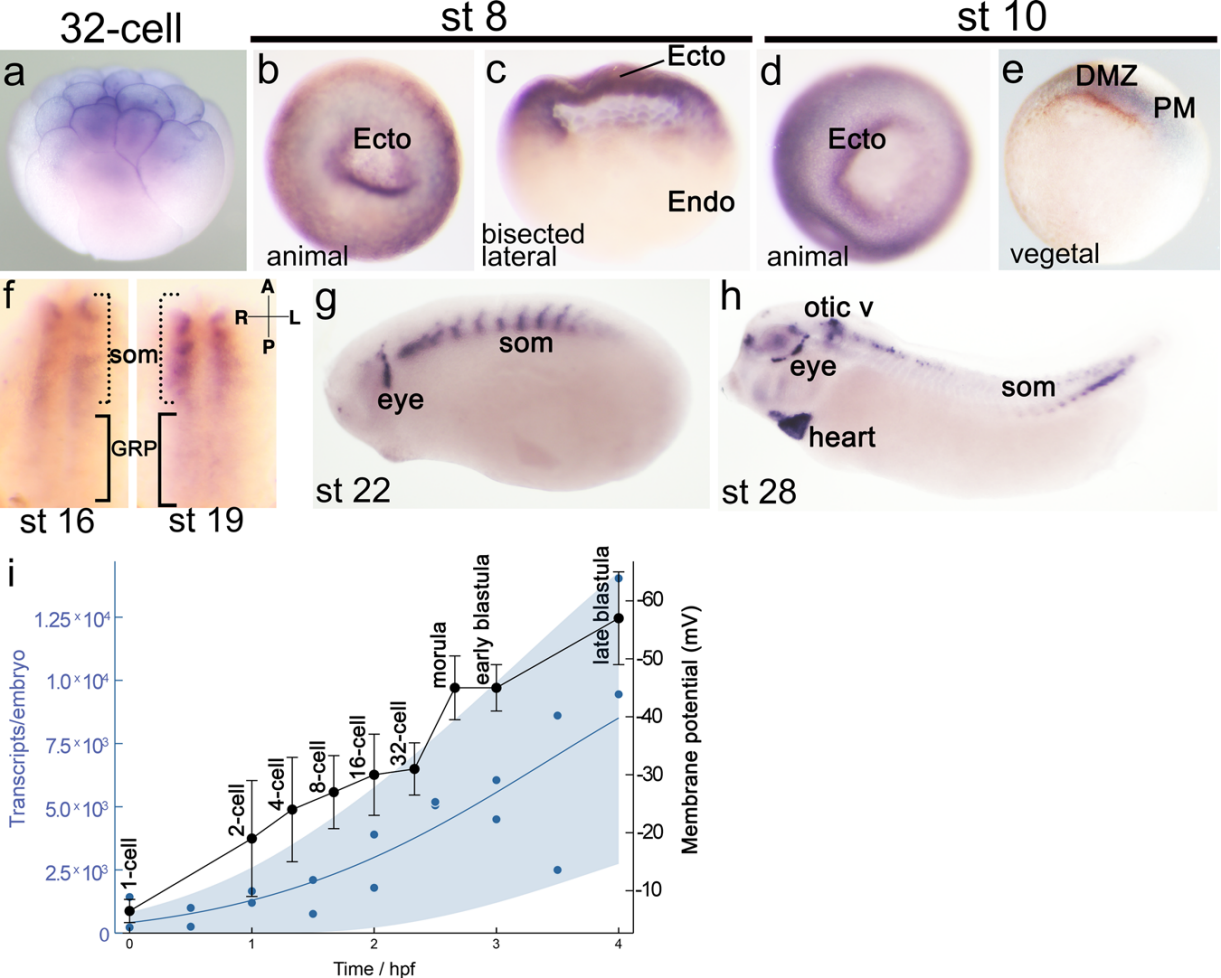
*kcnh6* expression analysis for *kcnh6* during *Xenopus* development. **a**–**h** *kcnh6* transcripts were detected by a full-length antisense probe via WMISH. Embryos and tissues displayed are in the following orientations: **a** animal pole to the top, **b** animal pole view **c** bisected with animal pole to the top, **d** animal pole view, **e** vegetal view and dorsal to the top, **f** gastrocoel roof plate with anterior to the top and vegetal view, **g**, **h** lateral view with anterior to the left and dorsal to the top; Ecto prospective ectoderm, Endo prospective endoderm, DMZ dorsal marginal zone (mesoderm), PM paraxial mesoderm, som somites, GRP gastrocoel roof plate. Representative images from *N* = 60 embryos (per developmental stage) over 3 independent experiments. **i** kcnh6 transcripts^19^ (blue) and V_m_^16^ (black) plotted during early *Xenopus* development. Blue dots represent mean transcript levels by temporal resolution RNA-seq, while blue shaded region marks Gaussian process 95% confidence interval of the data; *n* = 2 biological replicate time courses in original study. Membrane potential is presented as mean ± SD from at least *n* = 14 embryos in original study.

To address the question of when V_m_ is critical for embryonic development, we employed barium ions to block K^+^ channels at different time points of embryonic development, since K^+^ conductance is paramount for determining V_m_. Because K^+^ conductance drives the membrane voltage to a negative (hyperpolarized) potential, blocking K^+^ channels depolarizes cells. In line with the previous electrophysiological evidence demonstrating that embryos first become polarized at the blastula stage^16^, we found that Barium treatment affected embryonic development primarily when embryos were treated from blastula stages through gastrulation rather than at earlier cleavage stages (Fig. 1b, c, f, g, j–l)^17^. Embryonic development was affected in two ways: (1) 37 ± 3% (SEM) of the embryos failed to complete gastrulation (compared to just 4.7 ± 1% in control embryos) (Fig. 1b, c, j, k), and (2) 23 ± 2% that completed gastrulation exhibited misplacement of their organs relative to the left-right axis (compared to just 4 ± 2% in control embryos; Fig. 1f, g, j, l); these included abnormal heart looping to the left, an L-loop (vs a normal D-loop to the right), inverse gut rotation and misplacement of the gall bladder on the left (vs a normal positioning of the gall bladder on the right side of the body axis) (Fig. 1f, g). Because Barium can affect more than just K^+^ channels, we tested an alternative strategy for achieving membrane depolarization, namely manipulating V_m_ by increasing extracellular potassium ([K]_o_ in GHK eq. Fig. 1a). Increasing the extracellular potassium reduces the chemical driving force for potassium to leave the cell and decreases the potassium current which depolarizes the embryo (more positive V_m_). Incubating embryos in high K^+^ at blastula/gastrula stages was sufficient to cause a) gastrulation failure in 32 ± 6% of embryos (compared to just 6 ± 1% in controls Fig. 1b, d, k) and b) defective organ *situs* at later stages in 28 ± 3% of embryos (compared to just 4 ± 2% in controls; Fig. 1f, h, l). Thus, our results suggest that establishing proper V_m_ at blastula stages is essential for both gastrulation and LR patterning, providing context to previous work showing that V_m_ varies during embryonic development by becoming steadily more polarized from egg to blastula.

### KCNH6 is essential for LR patterning and gastrulation

Recent studies in patients with congenital heart disease identified a number of variants in KCNH ether-a-go-go (EAG) potassium channels (Table 1)^18^ as candidate disease genes. Many of these patients had heterotaxy, a disorder of LR development that can have a significant impact on the structure and function of the heart that can be life-threatening. While multiple ions can affect membrane potential, the flow of potassium down its electrochemical gradient (K^+^_in_>>K^+^_out_) has the largest impact on V_m_ because in most cell types cell membranes are most permeable to potassium. Since KCNH6 was the most common family member in a total of five patients with heterotaxy (Table 1), we began our studies by examining the CHD/Htx candidate gene, KCNH6.

**Table 1:** KCNH gene variants identified in patients with CHD

Blinded ID	Gene	Phenotype	Allele type	Class	AA change
1-09347	*KCNH1*	CTD/Htx	LOF het	splice	.
1-01004	*KCNH1*	CTD/Htx	LOF het	frameshift_deletion	p.G149fs
1-05070	*KCNH3*	LVO	LOF het	stopgain	p.R139X
1-07611	*KCNH3*	CTD	CmpHet	misD/misD	A911V/ S1021P
1-01856	*KCNH3*	LVO	CmpHet	misD misD	A357T/ F542L
1-05499	*KCNH3*	CTD	de novo	misD	p.E859D
1-02696	*KCNH5*	Htx/TGA	de novo	misD	p.N817S
1-05146	*KCNH6*	Htx	LOF het	stopgain	p.S858X
1-07078	*KCNH6*	CTD	LOF het	stopgain	p.E587X
1-02620	*KCNH6*	other	LOF het	stopgain	p.Q487X
1-06077	*KCNH6*	Htx	LOF het	frameshift_insertion	p.S671fs
1-02515	*KCNH6*	Htx	de novo	misD	p.T274M
1-01783	*KCNH7*	LVO	LOF het	stopgain	p.Y1162X
1-12888	*KCNH7*	CTD	LOF het	stopgain	p.E944X
1-06600	*KCNH8*	Htx/TGA	LOF het	frameshift_deletion	p.D782fs
1-06579	*KCNH8*	other	LOF het	stopgain	p.E576X

*CTD* conotruncal defect, *Htx* heterotaxy, *LVO* left ventricular outflow tract obstruction, *TGA* transposition of the great arteries, *LOF* loss of function, *CmpHet* compound heterozygote, *misD* damaging missense mutation according to previously published criteria.

**Fig. 3:**
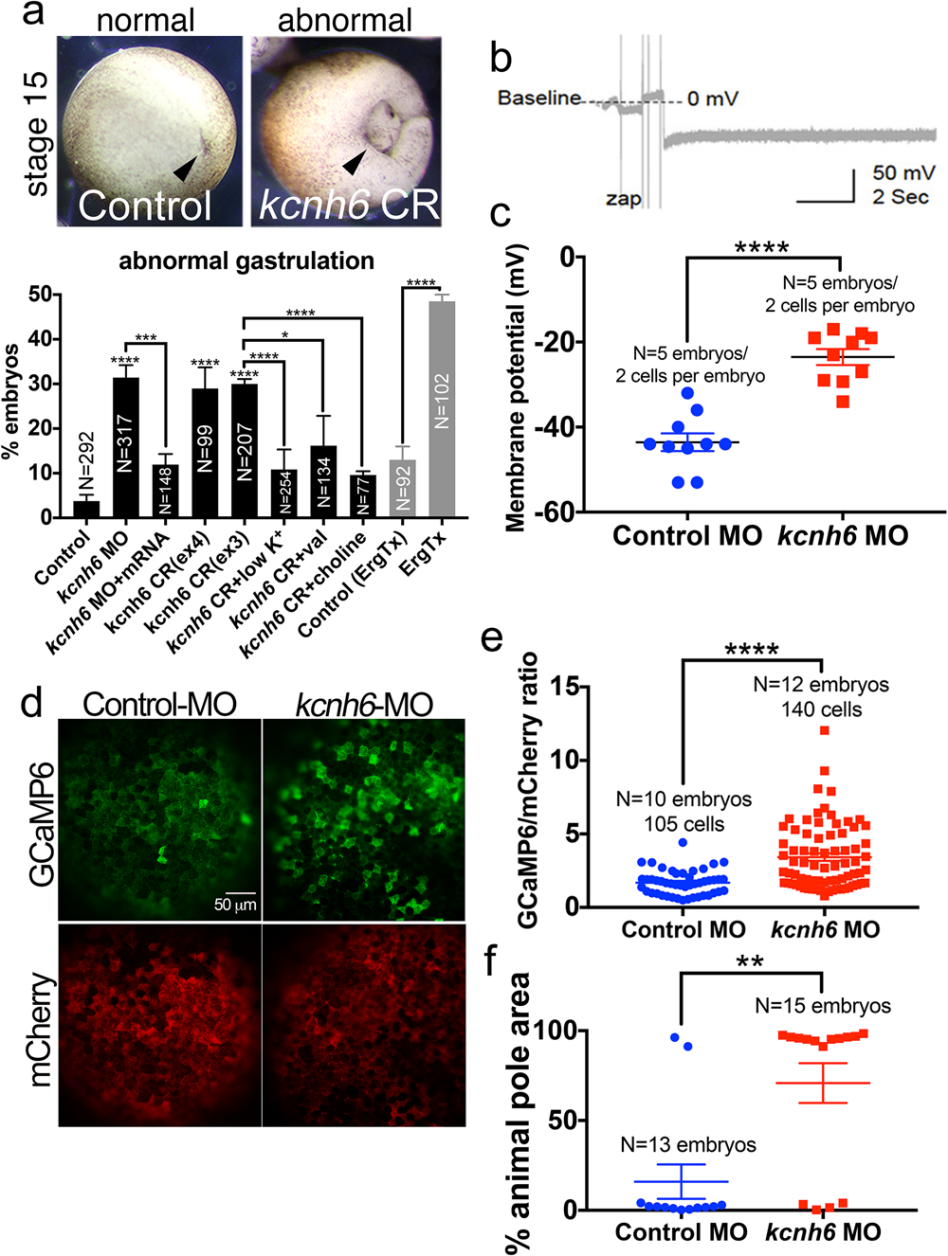
Membrane potential is important for gastrulation and regulates calcium levels at gastrulation onset. **a** Percentages of embryos with abnormal gastrulation after depletion of *kcnh6* (MO, CRISPR) or Kcnh channel blockade with Ergtoxin, and rescue of *kcnh6* depletion with medium conditions that hyperpolarize the V_m_ (low K^+^, val valinomycin, sodium substitution with choline; treatments performed stages 8–12). Above: examples of embryos scored for the graph; posterior views (dorsal to the top) of stage 15 embryos after successful (control) or unsuccessful (*kcnh6* CRISPR = CR) gastrulation; arrowhead points to blastopore closure. Graph reports mean ± SEM; total embryo numbers (N) in the graph are from 3 independent experiments (except for Ergtoxin: 2 independent experiments with devitellinized embryos); *p*-values are (*kcnh6*MO vs Control MO) = 8.66e–010, (*kcnh6*MO+mRNA vs *kcnh6*MO) = 2.59e-004, (*kcnh6*CRex4 vs Control) = 7.19e-018, (*kcnh6*CRex3 vs Control) = 5.79e-022, (*kcnh6*CR+low K^+^ vs *kcnh6*CR) = 1.64e-005, (*kcnh6*CR+val vs *kcnh6*CR+DMSO) = 1.22e-002, (*kcnh6*CR+choline vs *kcnh6*CR) = 2.34e-006, (ErgTx vs Control) = 6.57e-010; two-sided Fisher’s exact test. **b** Representative intracellular recording in the prospective ectoderm of a control stage 10 embryo; V_m_ is measured relative to the medium (baseline); the dip in membrane potential indicates the electrode breaking into the cell. **c** The V_m_ as measured by intercellular recordings in the prospective ectoderm of stage 10 Control MO and *kcnh6* MO-injected embryos; graph reports mean ± SEM; *p*-value (*kcnh6*MO vs Control MO) is 1.07e-006 (unpaired two-tailed student’s *t*-test); each data point represents one cell; data from 10 cells/5 embryos/3 independent experiments. **d** Live animal pole images of GCaMP6/mCherry at stage 10. **e** Quantification of GCaMP6 fluorescence intensity normalized to mCherry in mCherry+ cells; graph shows mean ± SEM; data points represent single cells; data from N cells (in graph)/10 embryos/3 independent experiments; *p* = 1.14e-006; unpaired two-tailed student’s *t*-test. **f** Maximum area undergoing simultaneous Ca^2+^ transients within a 20 s time lapse recording as a percentage of total animal pole area; the animal poles of 13 Control MO and 15 *Kcnh6* MO embryos were recorded over 3 independent experiments; *p* = 1.04e-003; unpaired two-tailed student’s *t*-test. Key for asterisks: *p ≤ 0.05, **p ≤ 0.01, ***p ≤ 0.001, ****p ≤ 0.0001, ns nonsignificant with *p* > 0.05. Source data are provided as a Source Data file.

In *Xenopus*, we found *kcnh6* to be expressed in the prospective ectoderm and dorsal/paraxial mesoderm at gastrulation onset, suggesting that it could play a role during gastrulation (Fig. 2a–h). Additionally, high temporal resolution RNA-seq shows that the increase in *kcnh6* transcripts parallels the trend of V_m_ polarization in the frog embryo (Fig. 2i)^16,19^. We thus tested a role for *kcnh6* in early embryonic development, and in determining V_m_ specifically. Two F0 CRISPRs independently targeting two different exons in *kcnh6* as well as a translation blocking morpholino oligo (MO) recapitulated the morphological defects observed in embryos depolarized with barium and high K^+^, including: (a) inability to complete gastrulation (Fig. 3a) (CRex3: 27 ± 4%; CRex4: 29 ± 5% and MO:31 ± 3% vs only 4 ± 1% in controls), and (b) abnormal organ *situs* (Supplementary Fig. 1a–h) (CRex3: 43 ± 12%; CRex4: 19 ± 1% and MO:91 ± 7% vs only 5 ± 3% in controls). Importantly, we established specificity of our depletion studies by the following criteria: (1) phenocopy with two non-overlapping sgRNAs via F0 CRISPR and one translation blocking MO (Fig. 3a and Supplementary Fig. 1a–h), (2) rescue of the MO phenotype with human *KCNH6* mRNA (Fig. 3a), and (3) detection of gene editing of the *kcnh6* locus by PCR amplification and Inference of CRISPR Edits (Supplementary Fig. 2)^20^. Moreover, treatment of blastula/gastrula embryos (stage 8 to stage 12) with Ergtoxin, a scorpion peptide that specifically acts as a pore blocker of the KCNH channel family^21^, also led to identical gastrulation and LR defects (Fig. 3a and Supplementary Fig. 1f–h). These results indicate that Kcnh channels, and specifically Kcnh6, contribute to gastrulation and LR development, consistent with their identification in patients with Htx/CHD.

### V_m_, rather than KCNH6 per se, is essential for gastrulation

Depletion/inhibition of potassium channels or elevation of extracellular K^+^ should lead to membrane depolarization. Thus, we reasoned that the inverse condition, namely hyperpolarizing by reducing extracellular K^+^, should rescue *kcnh6*-depleted embryos. Lowering extracellular K^+^ ([K]_o_ in GHK eq. Fig. 1a) increases the outward driving force for flow of K^+^, provided that other K^+^ channels are present. Indeed, lowering extracellular K^+^ rescues the gastrulation defect in *kcnh6-*depleted embryos (CRISPR and MO) (Fig. 3a and Supplementary Fig. 3a). To test the significance of K^+^ conductance independently of a specific K^+^ channel, we employed valinomycin, a K^+^ selective ionophore that inserts itself into the plasma membrane and mimics the ability of a K^+^ channel to passively conduct K^+^ down its electrochemical gradient. Application of valinomycin also rescued the gastrulation defect in *kcnh6-*depleted embryos (30 ± 1% gastrulation defect in *kcnh6* CR/DMSO- vs 16 ± 7% in *kcnh6* CR/Valinomycin-treated embryos) emphasizing the importance of K^+^ flux rather than a specific need for Kcnh6 itself or an alternative role of Kcnh6 in cell signaling (Fig. 3a and Supplementary Fig. 3a). Finally, to differentiate between membrane potential and an alternative role for K^+^ flux across the plasma membrane (e.g., cell volume regulation), as well as to unlink membrane potential from a specific conductance (i.e., potassium), we hyperpolarized by reducing extracellular Na^+^ and replacing it with equimolar choline. Choline has equivalent cationic charge to Na^+^ but cannot pass through channels and therefore does not influence the V_m_. Importantly, replacement of Na^+^ with equimolar choline does not affect the osmotic properties of the medium, ensuring that external Na^+^ depletion will only act on V_m_ and not on cell volume^22^. To determine the amount of sodium to replace with choline, we measured V_m_ using intracellular electrodes in the animal pole in the context of high K^+^ and rescue by choline substitution of sodium. Embryos exposed to high K^+^ were depolarized (V_m_ = −20 ± 4 mV) while embryos treated with high K^+^ and ½ sodium replacement with choline were repolarized to roughly the normal membrane potential (V_m_ = −41 ± 4 mV) (Supplementary Fig. 3b). Therefore, we tested ½ sodium replacement with choline on embryos and assayed for gastrulation which led to a remarkable rescue in *kcnh6*-depleted embryos (Fig. 3a). These data suggest that V_m_, which is determined by the conductance of K^+^ through Kcnh6 and influenced by other K^+^ and Na^+^ channels, is key to gastrulation.

Finally, we sought to measure the change in V_m_ when *kcnh6* is depleted. Using intracellular electrodes in the animal pole of *kcnh6* MO vs control MO-injected embryos at gastrulation onset, we recorded a V_m_ of −24 ± 1.9 mV in *kcnh6* MO vs −44 ± 2.1 mV in control MO embryos (Fig. 3b, c). Thus, Kcnh6 contributes ~20 mV to the cell’s negative resting potential, and embryos lacking *kcnh6* are abnormally depolarized compared to their control counterparts.

### Depolarized V_m_ increases calcium levels

We then asked how V_m_ is transduced into a signal that affects embryonic development. There are a limited number of voltage responsive elements in a cell. We reasoned that depolarization (V_m_ = −24 mV) in *kcnh6*-depleted embryos could aberrantly activate voltage-gated Ca^2+^ channels (VGCCs), which facilitate inward Ca^2+^ flux^23^. L-type VGCCs are present in the prospective ectoderm and dorsal mesoderm and can induce potent intracellular Ca^2+^ increases that can alter germ-layer patterning^24,25^, yet upstream regulators of these calcium channels remain elusive. Interestingly, intracellular Ca^2+^ is elevated after fertilization and during early cleavage stages but declines as the embryo approaches gastrulation^26^ concomitant with the onset of membrane polarization. We argued that, if VGCCs are aberrantly activated due to an abnormally depolarized V_m_, we should be able to detect changes in intracellular Ca^2+^ levels. To assess this, we microinjected the calcium indicator GCaMP6^27^ mRNA together with mCherry mRNA (to enable ratiometric analysis) into control MO or *kcnh6* MO embryos and performed calcium imaging in animal cells of early gastrula embryos. Within the animal pole of stage 10 control MO-injected embryos, we observed multiple intracellular calcium increases, signified by a pulse-like appearance of GCaMP6 fluorescence in isolated cells, which then propagated to adjacent cells (Supplementary Movie 1). These increases are well documented in *Xenopus* stage 8–12 gastrulae, i.e., last a few seconds, in which they spread to adjacent cells and then extinguish, are VGCC dependent and may contribute to neural induction^25,28^. We confirmed the existence of Ca^2+^ transients at stage 10 by performing 20 s time lapse recordings, and additionally observed that they are of low intensity and typically do not simultaneously affect more than 16 ± 10% of the total animal pole area (Supplementary Movie 1 and Fig. 3d–f). Interestingly, the same transients were dramatically increased in stage 10 *kcnh6* MO embryos both in intensity and area (Supplementary Movie 2 and Fig. 3d–f), affecting 71 ± 11% of the animal pole on average, with most embryos displaying simultaneous calcium increases in >90% of the animal pole. Thus, *kcnh6* contributes to a hyperpolarized V_m_ and is key for suppressing calcium levels at gastrulation onset, a signal that may facilitate correct gastrulation.

### Depolarization affects paraxial mesoderm and ectoderm

**Fig. 4:**
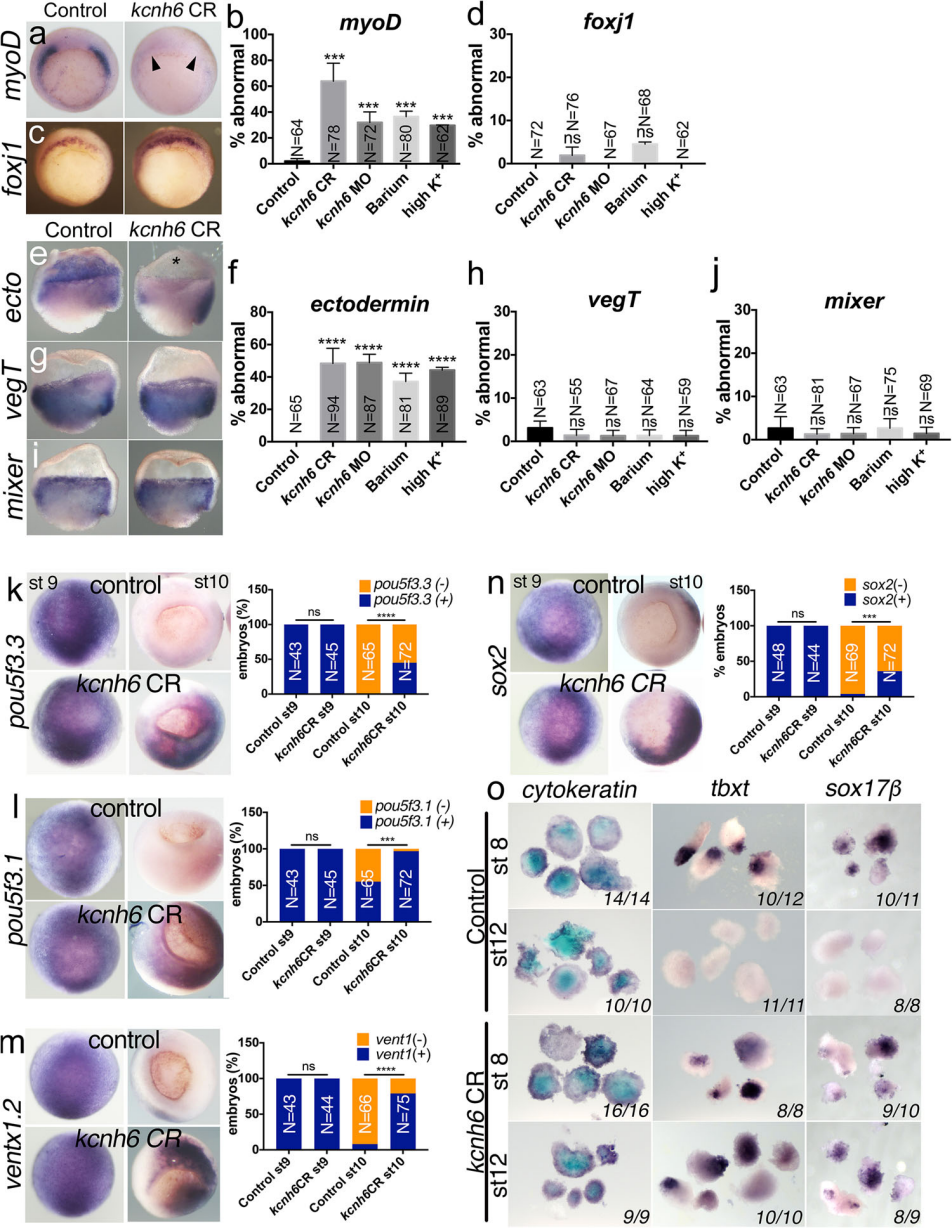
Membrane potential affects early gastrula patterning and pluripotency. **a**–**j** WMISH for germ-layer markers in early gastrula embryos (stage 10; **a**, **c**: vegetal views with dorsal to the top; **e**, **g**, and **i**: lateral view of bisected embryos, dorsal to right). Markers are for paraxial mesoderm (*myoD*), superficial dorsal mesoderm (*foxj1*), ectoderm (*ectodermin* = *ecto*), endoderm (*vegT*, *mixer*). Graphs (**b**, **d**) and (**f**–**j**) depict percentages of embryos with absent or strongly reduced expression of these markers; presented are mean ± SEM; total embryo numbers (N) are from 3 independent experiments; *p*-values (vs Control) are in **b**: (CR vs Control) = 1.61e-006, (MO vs Control) = 4.99e-003, (Ba^2+^ vs Control) = 4.45e-003, (high K^+^ vs Control) = 7.56e-004, and in **f**: (CR vs Control) = 6.81e-013, (MO vs Control) = 2.7e-013, (Ba^2+^ vs Control) = 1.58e-009, (high K^+^ vs Control) = 6.61e-012; ns (nonsignificant) for *p* > 0.05; two-sided Fisher’s exact test. **k**–**n** WMISH for *Xenopus* pluripotency genes *pou5f3.3*, *pou5f3.1*, *ventx1.2*, and *sox2* at stages 9 and 10 (animal pole views). Graphs (**k–n**) depict mean percentages of embryos with present (+/blue) or absent (−/orange) gene expression; total embryo numbers (N) are from 3 independent experiments; p-values (vs Control) are in **k**: *p* = 2.01e-008, in **l**: *p* = 1.52e-010, in **m**: *p* = 6.73e-018 and in **n**: *p* = 1.85e-006; ns (nonsignificant) for *p* > 0.05. Key for asterisks in all graphs: *p ≤ 0.05, ***p ≤ 0.001, ****p ≤ 0.0001, ns nonsignificant. **o** Differentiation potential of animal caps excised at stage 8 or 12 and treated with no activin (differentiation into epidermis marked by cytokeratin), low activin (differentiation into mesoderm marked by tbxt) and high activin (differentiation into endoderm marked by sox17*β*). In each image, the numbers on the bottom right report caps with the indicated phenotype vs total number of caps analyzed over 2 independent experiments. Source data are provided as a Source Data file.

For gastrulation to proceed normally, two steps are critical: first, the germ layers of the blastula embryo (ectoderm, mesoderm, and endoderm) must be patterned correctly and second, the embryo must undergo the cellular rearrangements that drive morphogenesis. Calcium has been previously identified to play a role in morphogenesis cell behaviors during gastrulation^29,30^. Alternatively, calcium may play a role in patterning the mesoderm that also drives gastrulation cell movements. Patterning precedes morphogenesis, and morphogenesis can fail as a result of abnormal patterning. We, therefore, first examined if patterning is disrupted in V_m_-depolarized embryos via marker gene expression. Since the mesoderm is critical for gastrulation movements, we began with this germ layer. Markers of the dorsal (*gsc*, *nodal3*) and ventral mesoderm (*vent2*) appeared unaffected in *kcnh6-*depleted, barium and high K^+^ depolarized embryos (Supplementary Fig. 4e–j); however, the paraxial mesoderm fates appeared lost as marked by *myoD*, *myf5*, and *tbxt* (*brachyury*, *xbra*) (Fig. 4a–d and Supplementary Fig. 4a–d). In fact, absent patterning of paraxial mesoderm by *myoD* persisted into the Left-Right Organizer (Supplementary Fig. 5), a transient structure formed at the end of gastrulation where cilia driven flow is thought to pattern the LR axis^31,32^. In the LRO, *dand5* (*coco*) is normally expressed in the paraxial mesoderm symmetrically until cilia driven flow suppresses *dand5* expression on the left^33,34^. However, consistent with a mispatterning of the paraxial mesoderm in the LRO, *dand5* was also absent even before the occurrence of cilia driven flow (Supplementary Fig. 1i, j). A disruption in the LRO is further supported by defective *pitx2c* expression in the left lateral plate mesoderm at later stages (Supplementary Fig. 1k, l). Therefore, in *kcnh6*-depleted embryos, the paraxial LRO is mispatterned, and a defect in this tissue can be detected already at the onset of gastrulation.

When certain biochemical signaling factors are depleted, loss of one-cell fate (e.g., paraxial mesoderm) is often concomitant with gain of another cell fate^35–37^. Since the dorsal or ventral mesoderm appeared unaffected (Supplementary Fig. 4e–j), we considered that the ectoderm or endoderm might be expanded into the mesodermal area of embryos with abnormally depolarized V_m_^38,39^. Interestingly, while the endoderm (*vegT*) and its border to the mesoderm (*mixer*) seemed unaffected (Fig. 4g–j), ectodermal fates (*ectodermin* and *foxI1a*) were lost similar to the paraxial mesoderm (Fig. 4e, f and Supplementary Fig. 4k, l. In fact, depletion of *ectodermin* (*trim33*) leads to developmental arrest midway through gastrulation^39^, which corresponds to the arrested phenotype in a portion of depolarized embryos. These results indicate that V_m_ has an effect on germ-layer differentiation, and specifically paraxial mesoderm and ectoderm, at gastrulation onset (Fig. 4a–j).

### Cacna1c responds to V_m_ depolarization

**Fig. 5:**
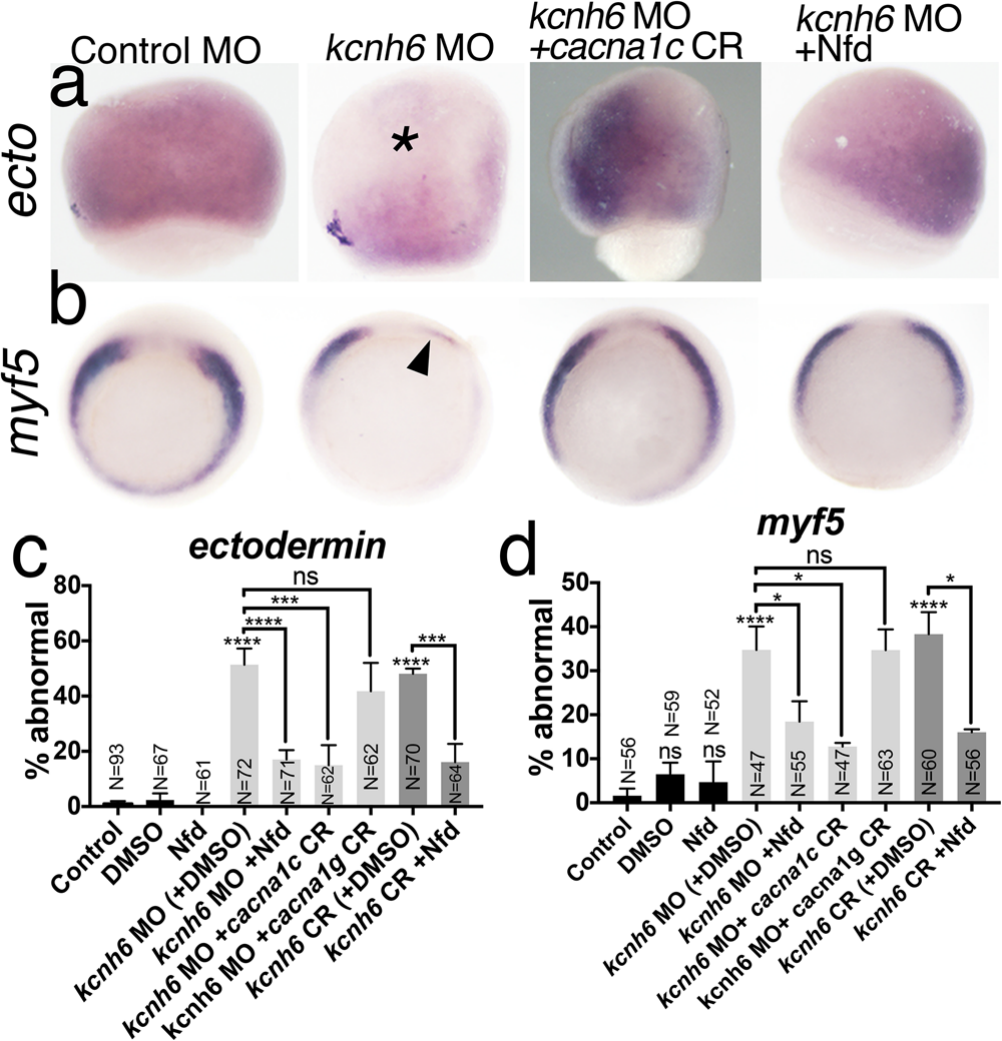
The role of VGCCs in germlayer differentiation. **a** WMISH for *ectodermin*; lateral views with the animal pole to the top; asterisk marks the animal pole with loss of *ectodermin* expression; embryos with absent expression are quantified in **c**; CR CRISPR, Nfd nifedipine. **b** WMISH for *myf5:* vegetal views with dorsal to the top; arrowhead marks loss of expression; embryos with abnormal expression are quantified in **d**. **c**, **d** Percentages of embryos with abnormal *ectodermin* (**c**) and *myf5* (**d**) expression. Graphs depict mean ± SEM; *p*-values are in **c**: (*kcnh6*MO+DMSO vs DMSO) = 2.88e-006, (*kcnh6*MO+Nfd vs *kcnh6*MO+DMSO) = 4.59e-002, (*kcnh6*MO+*cacna1c*CR vs *kcnh6*MO) = 1.53e-002, (*kcnh6*MO+*cacna1g*CR vs *kcnh6*MO) > 9.99e-001 (ns), (*kcnh6*CR+DMSO vs DMSO) = 3.50e-007, (*kcnh6*CR+Nfd vs *kcnh6*CR+DMSO) = 1.20e-002; in **d**: *p*-values are (*kcnh6*MO+DMSO vs DMSO) = 4.07e-015, (*kcnh6*MO+Nfd vs *kcnh6*MO+DMSO) = 8.92e-005, (*kcnh6*MO+*cacna1c*CR vs *kcnh6*MO) = 4.78e-005, (*kcnh6*MO+*cacna1g*CR vs *kcnh6*MO) = 2.24e-001 (ns), (*kcnh6*CR+DMSO) = 2.04e-014, (*kcnh6*CR+Nfd) = 1.45e-003; two-sided Fisher’s exact test; total embryo numbers (N) in the graphs are from at least 2 independent experiments; Key for asterisks: *p ≤ 0.05, ***p ≤ 0.001, ****p ≤ 0.0001, ns nonsignificant for *p* > 0.05. Source data are provided as a Source Data file.

In depolarized embryos, we have established (1) changes in cell fate and (2) elevated intracellular calcium levels, so we next tested if these aberrant cell fates are dependent on voltage-gated calcium channels. To determine the specific embryonic VGCCs downstream of V_m_, we reviewed our available high temporal resolution RNA-Seq data^19^. *Xenopus* contains detectable transcripts of L- and T-type VGCCs between the 1-cell and gastrula stages, while other VGCC types (N-, R-, and P/Q) are less abundant. L-type VGCCs become activated at V_m_ > −40 mV (and then inactivated at V_m_ > 10 mV) and have been previously implicated in gastrula patterning^23,24,29^ while T-type channels become inactivated at V_m_ > −60 mV and would be inactive both at physiological V_m_ (~−50 mV) and at more depolarized potentials. Therefore, we tested the L-type VGCC blocker nifedipine. This significantly ameliorated both *ectodermin* (ectoderm) and *myf5* (paraxial mesoderm) expression losses in *kcn6* knockdown embryos (Fig. 5a–d). Specifically, *myf5* was lost only in 16 ± 1% and 18 ± 5% of nifedipine-treated *kcnh6* CR and MO embryos (vs 38 ± 5% and 35 ± 5% in *kcnh6*CR or MO embryos treated only with DMSO, Fig. 5b, d). Similarly, absent *ectodermin* was only observed in 16 ± 7% and 17 ± 3% of nifedipine-treated *kcnh6* CR and MO embryos (vs 48 ± 2% and 51 ± 6% in *kcnh6* CR or MO embryos treated with DMSO, Fig. 5a, c). Of the VGCCs identified in our RNA-seq data at blastula/gastrula stages, two genes *cacna1c* (Cav1.2; L-type) and *cacna1g* (Cav3.1; T-type) encode alpha (pore-forming) channel subunits, which are indispensable for channel function. Co-depletion of *cacna1c* in *kcnh6-*depleted embryos rescued expression of *ectodermin* and *myf5*, while co-depleting *cacna1g* (Fig. 5a–d) resulted in no rescue. Thus, Kcnh6, which sets a negative V_m_, is essential to limit the activation of L-type VGCCs and specifically Cacna1c, a critical step for ectodermal and paraxial mesodermal differentiation.

### V_m_ depolarization maintains pluripotency in gastrula embryos

The loss of some cell fates (ectoderm and paraxial mesoderm) without a concomitant expansion of other cell fates was puzzling given that most biochemical signaling factors (Wnt, BMP, Nodal) generally balance different cell fates in the early embryo. We speculated that these unspecified cells may simply lack the ability to assume *any* cell fate because they remain pluripotent abnormally. To test this hypothesis, we examined markers of pluripotency *OCT4*, *NANOG*, and *SOX2*. In *Xenopus*, there are three *OCT4* homologs (*pou5f3.1, 2,* and *3, formerly oct91*, *oct25*, and *oct60*)^40^, and the *ventx1.2/2.2* factors, which have overlapping functions in maintaining differentiation competence and are thought to be structurally and functionally equivalent to mammalian Nanog^41^. *Sox2*, a core pluripotency factor in mammals, is highly conserved in amphibians and also expressed at high levels prior to lineage commitment throughout the *Xenopus* blastula^42,43^. We examined the prospective ectoderm of embryos, which is best characterized in its pluripotent properties, and confirmed that *pou5f3.1, pou5f3.3*, *sox2*, and *ventx1.2* are robustly expressed at stage 9 prior to lineage commitment, but their transcripts are sharply reduced by stage 10 in control embryos (Fig. 4k–n). In contrast, *kcnh6* CR embryos retain robust expression of these factors well beyond stage 9 and into stage 10, a prolonged expression compared to wildtype embryos (Fig. 4k–n). This, in turn, is not due to a general delay in development, since *kcnh6* CR embryos were staged according to the physical progression of gastrulation, i.e. presence of blastopore lip. Moreover, in *kcnh6*-depleted late gastrula embryos, abnormal maintenance of *pou5f3.3* and *ventx1.2* can be abolished by incubating the embryos in L-type VGCC blocker nifedipine (Supplementary Fig. 6b–d). These results suggest that *kcnh6* is upstream of V_m_ and VGCCs in promoting the exit from pluripotency, which takes place as gastrulation proceeds.

Based on this result, we sought to test the pluripotency of these *kcnh6*-depleted embryos. In the blastula (stage 9), the prospective ectoderm or “animal cap” contains cells that when explanted will differentiate into epidermis (Fig. 4o). Importantly, when stage 8–9 explanted animal cap cells are treated with activin, they can be differentiated into mesodermal and endodermal cell fates indicating that they are pluripotent (Fig. 4o), an assay used for decades to test the activity of a host of differentiation factors. However, towards the end of gastrulation at stage 12, these animal cap cells are no longer pluripotent and when explanted will only differentiate into epidermis, even when stimulated with activin^43,44^ (Fig. 4o). Using this animal cap assay, we sought to test the role of *kcnh6* in determining pluripotency. We explanted stage 9 and stage 12 animal caps and assayed differentiation under three conditions: (1) no activin to examine spontaneous differentiation into epidermis (marked by *cytokeratin*), (2) low activin to stimulate differentiation into mesoderm (*tbxt*), and (3) high activin to stimulate differentiation into endoderm (*sox17β*). Both control and *kcnh6* CR animal caps explanted from stage 9 embryos were capable of differentiating into all three germ layers, indicating full differentiation potential even when *kcnh6* is depleted (Fig. 4o). On the other hand, as expected, animal caps explanted from stage 12 control embryos differentiated into epidermal fate but not into meso- or endoderm despite activin administration (Fig. 4o). Strikingly, stage 12 animal caps explanted concurrently from *kcnh6*-depleted embryos were able to differentiate into cell fates of all three germ layers with activin administration (Fig. 4o). From these and the previous experiments, we conclude that V_m_ polarization via *kcnh6* enables the exit from pluripotency.

### V_m_ limits mTOR to allow for exit from pluripotency

**Fig. 6:**
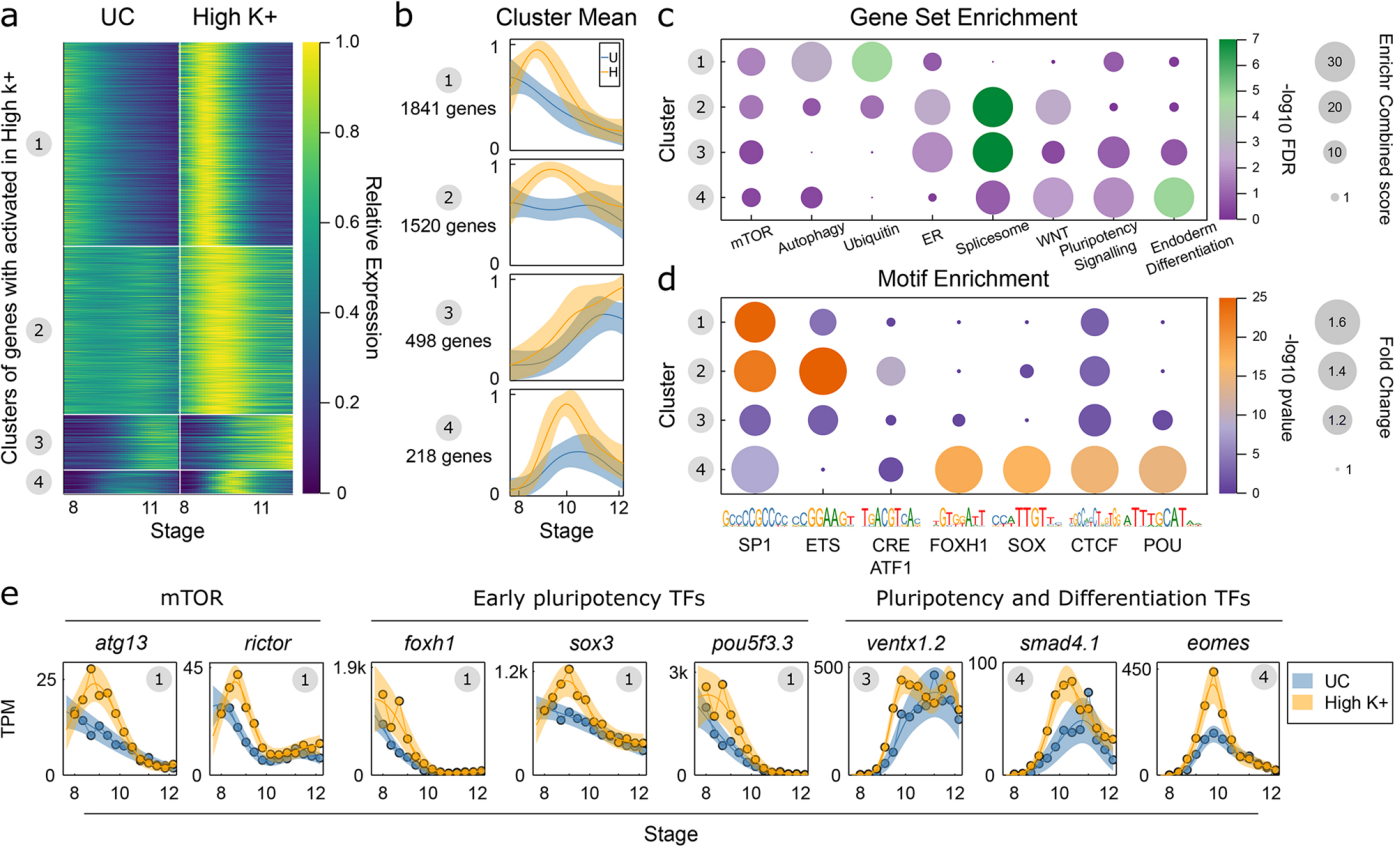
High-resolution temporal RNA-seq identifies mTOR and Ca^2+^ Gene Regulatory Network. **a**, **b** Summary of activated gene clusters by **a** heatmap and **b** cluster average. Data is Gaussian process median for each gene normalized by maximal value, shaded region in **b** is ±1 SD for each cluster. UC untreated control; High K^+^ (=depolarizing conditions). **c** Bubble plot for selection of gene set enrichment terms, calculated with Enrichr, see Methods for definition of terms, and Supplementary Data 1 for full set of enrichments. Bubble size reflects Enrichr Combined score and color indicates −log10 FDR. **d** Bubble plot enrichment of TF motifs in 500 bp upstream of cluster promoters, see also Supplementary Fig. 7g. Bubble size reflects fold change over background and color is −log10 Hypergeometric right tail *p*-value for enrichment. **e** Expression of exemplar genes in control and high K+. Central line and shaded region are transformed Gaussian process median and 95% CI. Circle in top right hand corner gives cluster number. UC (=untreated control), High K^+^ (=depolarizing conditions). Data analysis performed from *N* = 13 samples/each 10 embryos over one biological replicate time course.

Our findings indicate that a polarized V_m_ limits voltage-gated calcium channels and intracellular calcium, a process that reduces the expression of pluripotency genes as germ-layer differentiation initiates. A critical question is what are the signaling pathways invoked when V_m_ is depolarized or intracellular calcium is elevated. To address this question in an unbiased manner, we temporally profiled gene expression via RNA-Seq in control and high K^+^ depolarized embryos by collecting embryos every 30 min from pre- to post-gastrula stages (stages 8–12; Supplementary Fig. 7a–d). We identified genes exhibiting temporal differential expression employing a Gaussian Process framework^19^; this determines genes whose expression trajectory differs between control and high K^+^ embryos over the time course. We found 4043 genes on average activated in high K^+^ over the time course, and 1101 genes on average repressed (Supplementary Fig. 7e). We further used k-means clustering to subdivide these into 8 clusters, 4 activated (A1–4, Fig. 6a, b) and 4 repressed (R1-4, Supplementary Fig. 7f, g). In both cases, the clustering segregated genes showing dysregulated gene expression prior to gastrulation (Clusters 1, 2) and during gastrulation (Clusters 3,4) (Fig. 6a, Supplementary Fig. 7f, g). To assess the composition of these clusters, we performed gene set enrichment using Enrichr^45^. Comparing activated clusters to repressed clusters over seven different annotated gene set libraries, we found 1279 terms significantly associated with at least one of the 4 activated clusters, but only 44 terms significantly associated with repressed clusters. Therefore, given the total number of genes and associated terms, we focused our attention on the analysis of the activated genes.

We found a hierarchy of gene set enrichments from early to late in our time course, reflecting the changing response in the transcriptome (Fig. 6c). Notably, we found enrichment for the mTOR signaling pathway activated early with members including *rictor, depdc5, pik3cb, stk11, atg13* in cluster A1 (Fig. 6e) and *akt1s1, gsk3b, lamtor1/2* in cluster A2. The enrichment for mTOR members continues to span all activated clusters (FDR < 3 × 10^−5^ over all activated clusters combined). This enrichment is accompanied by pathways associated with mTOR, including autophagy, ubiquitin transferase activity, and ER response (Fig. 6c). The intermediate clusters A2 and A3 show the most prominent ER response enrichment together with significant upregulation of spliceosome machinery (including 12 snRNPs and 5 SRSF family members). In contrast, in later clusters, we find enrichments for terms explaining the sustained pluripotency and germ-layer defects, including pluripotency (*pou5f3.1, pou5f3.2, ventx1.2*) and WNT signaling (*fzd7*, *wnt8a, tcf7l1*) (Fig. 6c, e). Key members of the *Xenopus* pluripotency network are also found activated early, in cluster A1 including *foxh1, sox3*, and *pou5f3.3* (Fig. 6c, e).

To build the underlying gene regulatory networks, we examined transcription factor motif enrichment in the promoters of each of these activated gene clusters. Mirroring the gene set enrichments, we found motif enrichments segregated between early (A1,2) and later (A3,4) gene clusters (Fig. 6d, Supplementary Fig. 7h). We find strong enrichment for SP1, ETS, YY1, RFX, and CRE/ATF1 motifs driving gene expression changes in clusters A1 and A2. Interestingly, both ETS and CRE motifs are bound by factors responsive to calcium. Calcium induced phosphorylation of ETS1 inhibits binding activity^46^, and CRE elements are bound by calcium responsive family members, CREB1, CREM, and ATF1^47–49^. In our data, each of these factors are expressed at high levels at stage 8, and then are gradually downregulated to a minimum at stage 12. Of note, their mRNAs are not upregulated in high K^+^ conditions (Supplementary Fig. 7i). This suggests that the activity of these factors is post-translationally modified in depolarized embryos experiencing high Ca^2+^ to drive gene expression changes. In the case of ETS1, this factor may act to repress gene expression in normal germ-layer resolution and this repression is removed in high Ca^2+^ embryos. Supportive of these factors driving gene expression, we find a large intersection between activated gene clusters A1 and A2 and genes found in proximity to publicly available ETS1, CREB1 and CREM binding sites (Supplementary Fig. 7j); in the case of activated cluster A2 this remarkable enrichment accounts for 794/1520 (52.2%) of genes found in proximity to one of ETS1, CREB1, and CREM (FDR < 10^−26^,10^−23^,10^−35^, respectively, Fisher Exact Test). Therefore, we find that initial transcriptional responses captured by activated gene clusters A1 and A2 appear to be largely driven directly by calcium response. This includes the activation of mTOR and pluripotency genes in high K^+^ conditions; mTOR genes show an enrichment in CRE sites in their promoters across all clusters (*p* < 0.0072, Odds Ratio 2.46, Fisher’s Exact Test), pluripotency genes show enrichment for CRE sites in cluster A1 and A2 (*p* < 0.017, Odds Ratio 3.08, Fisher’s Exact Test), and ETS sites in cluster A2 (*p* < 0.021, Odds Ratio 5.15, Fisher’s Exact Test).

Turning to the genes activated later, particularly, those associated with pluripotency and germ-layer commitment in cluster A4, we find comprehensive enrichment of FOXH1, SOX and POU motifs in their promoters (Fig. 6d). These motifs correspond precisely with the early pluripotency TFs whose transcripts are activated in cluster A1 (Fig. 6e). Together, our high-resolution temporal profiling of the transcriptome in control and high K^+^ conditions reveals a cascade of transcriptional activation. We propose a model where a depolarized membrane opens VGCCs and elevates intracellular calcium leading to the expression of transcripts (including mTOR and pluripotency factors) whose promoters are enriched with calcium responsive motifs. This is followed by the activation of transcripts involved in pluripotency and germ-layer commitment, driven by the pluripotency factors activated in the early wave of gene expression.

**Fig. 7:**
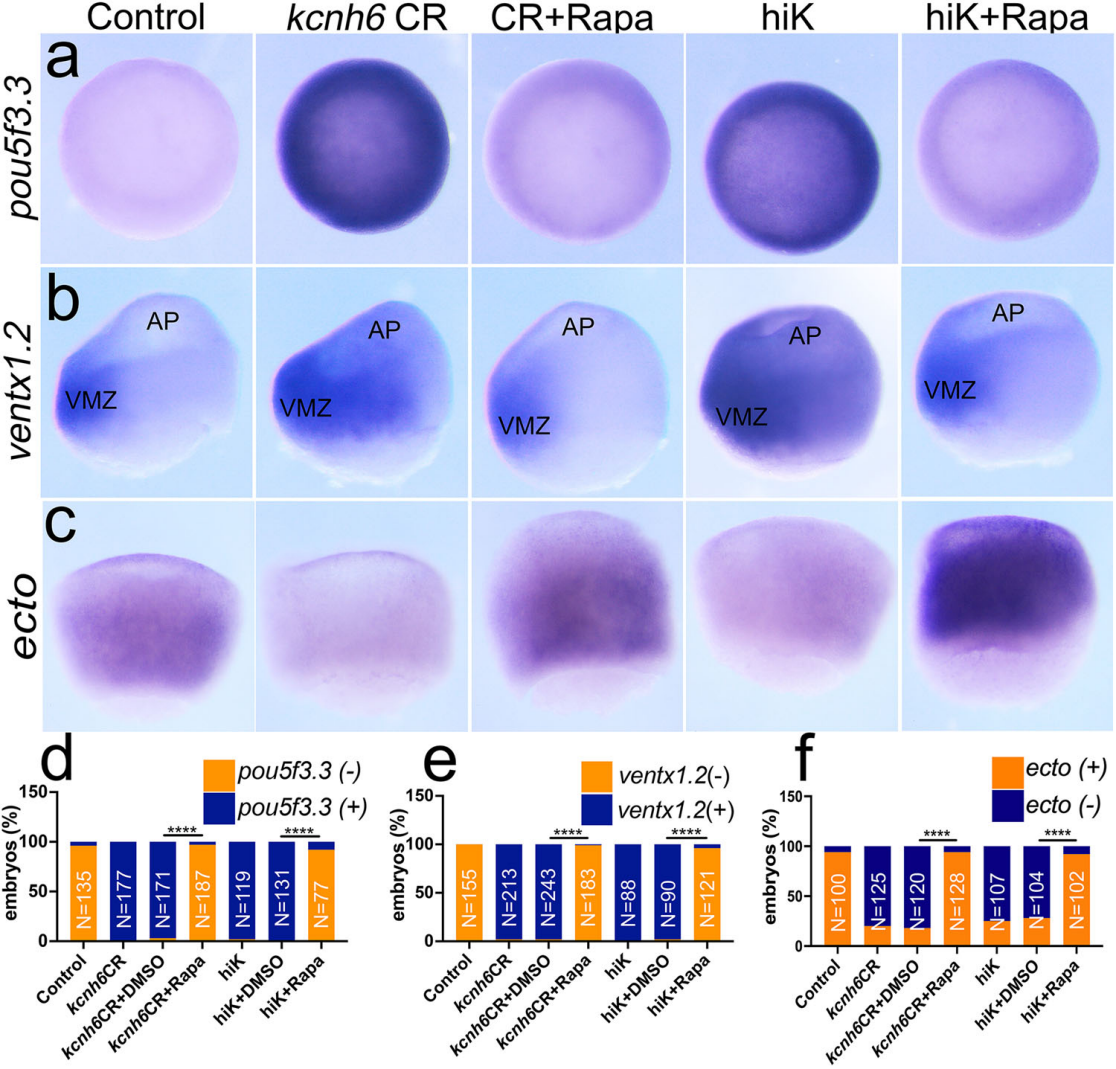
V_m_ polarization limits mTOR which promotes pluripotency. **a**–**c** WMISH for pluripotency markers *pou5f3.3, ventx1.2,* and differentiation marker *ectodermin* in stage 10 embryos, depolarized by *kcnh6* depletion (*kcnh6*CR) or high K+ (hiK), and treated with vehicle (DMSO) or rapamycin (Rapa) as indicated; views are **a** animal pole; **b**, **c** lateral view with dorsal to the right; AP animal pole, VMZ ventral marginal zone. **d**–**f** Quantification of stage 10 embryos with present (+) or absent (−) expression of markers *pou5f3.3* (**d**)*, ventx1.2* (**e**), and *ectodemin* (**f**) in the animal pole area. *p*-values in **d** are: (*kcnh6*CR+Rapa vs *kcnh6*CR+DMSO) = 3.34e-087 and (hiK+Rapa vs hiK+DMSO) = 2.89e-047, in **e**: (*kcnh6*CR+Rapa vs *kcnh6*CR+DMSO) = 1.60e-110 and (hiK+Rapa vs hiK+DMSO) = 1.93e-050, and in **f**: (*kcnh6*CR+Rapa vs *kcnh6*CR+DMSO) = 3.38e-040 and (hiK+Rapa vs hiK+DMSO) = 4.89e-02; two-sided Fisher’s exact test; total embryo numbers (N) in the graphs were collected over 3 independent experiments for *pou5f3.3* and *ventx1.2* and over 4 independent experiments for *ectodermin*; key for asterisks: ****p ≤ 0.0001. Source data are provided as a Source Data file.

Our transcriptome analysis not only revealed a potential gene regulatory network but pointed towards a role for mTOR. mTOR is critical for multiple cellular processes including autophagy, nutrient sensing, and an emerging role in pluripotency^50–56^. Because the expression of mTOR pathway members was increased in depolarizing conditions and pathways associated with mTOR, we reasoned that mTOR signaling was upregulated and maintained pluripotency in these depolarized embryos. To test this hypothesis, we applied the mTORC1 inhibitor, rapamycin, to depolarized gastrulating embryos to see if this could abolish the aberrant expression of pluripotency markers *pou5f3.3* and *ventx1.2* in the animal pole and activate germ-layer differentiation. Rapamycin dramatically lowered expression of *pou5f3.3* and *ventx1.2* in the animal pole in *kcnh6* CR and high K^+^ treated embryos compared to those embryos treated with vehicle alone and appeared comparable to untreated control embryos (Fig. 7a, b, d, e). Conversely, the expression of the ectodermal marker, *ectodermin*, which was reduced in depolarizing conditions (*kcnh6* depletion or exposure to high K^+^), was recovered with rapamycin treatment (Fig. 7c, f). Therefore, a polarized V_m_ at gastrulation onset is critical for limiting mTOR in order to suppress pluripotency genes and enter differentiation.

**Fig. 8:**
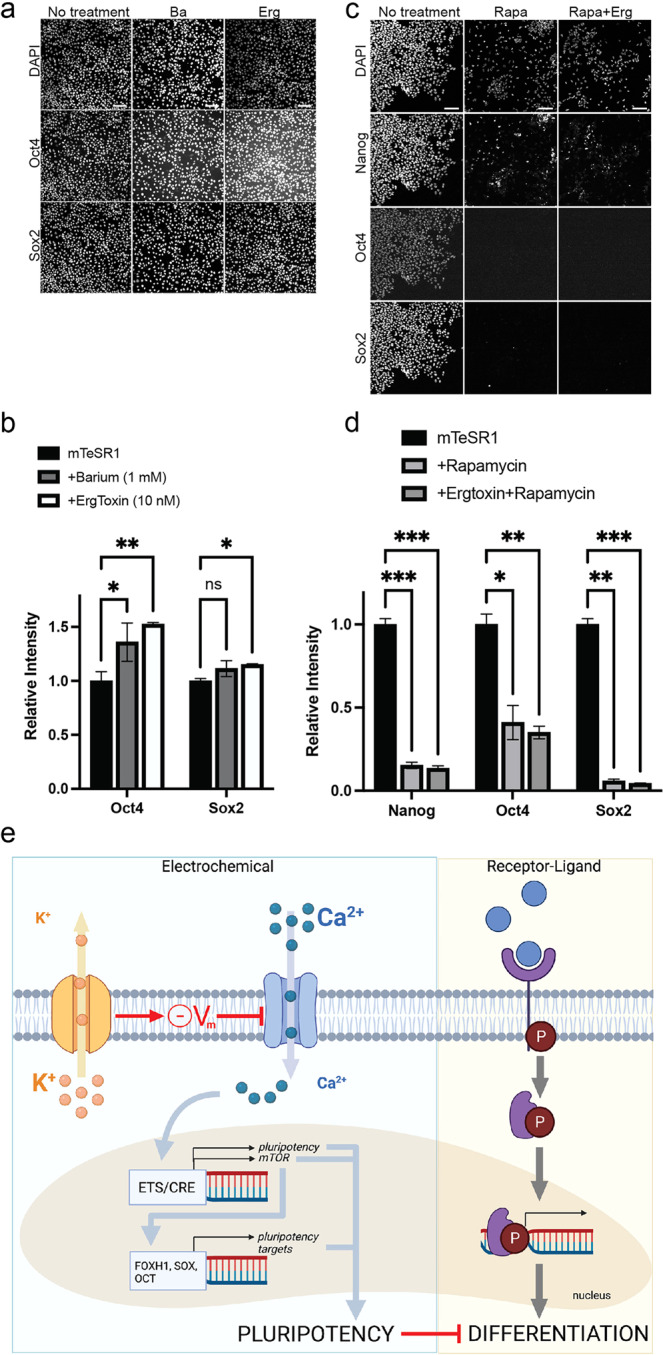
Potassium channels affect pluripotency in hESCs. **a** Images showing untreated hESCs grown in mTeSR1 media or cells treated with 1 mM Barium or 25 nM Ergtoxin and immunostained for pluripotency factors. Scale bar = 100 μm. **b** Quantification of the results in **a**; AU arbitrary units. *p*-values are Oct4: (control vs Ba) = 0.03, (control vs Erg) = 4.5e-04; Sox2 (control vs Ba) = 0.06, (control vs Erg) = 4.4e-04. **c** Images showing untreated hESCs grown in MEF-CM media or cells treated with 100 nM rapamycin with or without 25 nM Ergtoxin. Scale bar = 100 μm. **d** Quantification of the results in **c**; AU arbitrary units. *p*-values are Nanog: (control vs Rapa) = 4.5e-04, (control vs Rapa+Erg) = 4.3e-04; Oct4: (control vs Rapa) = 0.002, (control vs Rapa+Erg) = 0.002; Sox2 (control vs Rapa) = 7.7e-04, (control vs Rapa+Erg) = 7.6e-04. Graphs (**b**) and (**d**) present mean ± SEM; key for asterisks: *p ≤ 0.05, **p ≤ 0.01; ***p ≤ 0.001; ns nonsignificant for *p* < 0.05; data were derived from 400 cells/3 independent replicates over at least 2 independent experiments. Source data are provided as a Source Data file. **e** Model for the onset of embryonic differentiation depicting classical biochemical signaling (right) that is complemented by regulation via membrane potential (left). In the electrophysiological pathway, potassium channels set the membrane potential, which limits activation of voltage-gated calcium channels and suppresses intracellular Ca^2+^ levels. Both pathways result in changes in gene expression, mediated by intracellular signal transducers (right), or by factors that require calcium (left). While biochemical pathways are essential to induce expression of differentiation factors, the electrophysiological pathway affects cell fate indirectly by controlling the timing of downregulation of pluripotency genes. Adapted from “Transporters”, by BioRender.com (2022). Retrieved from https://app.biorender.com/biorender-templates.

Finally, we tested whether our findings would also apply to human embryonic stem cells (hESCs). At stage 9, *Xenopus* animal cap cells are pluripotent in that they can, under appropriate conditions, form derivatives of any of the three germ layers (Fig. 4o). However, they contrast with hESCs in their limited capacity for self-renewal as the brisk pace of embryonic development proceeds. Therefore, we turned our attention to hESCs to test our findings in the context of a self-renewing pluripotent state and to determine their relevance to human development. hESCs are already highly pluripotent, and we wondered if depolarization would lead to elevations in the pluripotency markers OCT4 and SOX2. Indeed, hESCs grown for two days with Ergtoxin to specifically block KCNH channels showed a modest but significant elevation of these markers over their already high levels in the pluripotency state as indicated by immunostaining and qPCR against these markers (Fig. 8a, b, Supplementary Fig. 8a, b). While not as specific as Ergtoxin for KCNH channels, Barium showed similar trends but did not rise to statistical significance. qPCR for the markers *OCT4*, *SOX2*, and *NANOG* also revealed upregulation of these genes at the transcript level, with *OCT4* and *SOX2* upregulated by day 2 and all three genes upregulated on day 5 (Supplementary Fig. 8a, b).

We also tested whether blocking K^+^ channels with Ergtoxin affected the kinetics of differentiation. BMP4 induces differentiation to either mesodermal or extraembryonic fates in a dose-dependent manner^57–59^. Ergtoxin caused a significant delay in downregulation of pluripotency markers such as SOX2 at 12 h with a similar trend in NANOG (Supplementary Fig. 8c, d). As in *Xenopus*, the timing of differentiation of human embryonic stem cells appears significantly affected under depolarizing conditions.

To test whether the role of mTOR signaling downstream of membrane depolarization is conserved, we treated hESCs with rapamycin with or without Ergtoxin. Treatment with rapamycin led to reduction of pluripotency markers in a dose-dependent manner with near complete loss by 5 days (Fig. 8c). In the presence of rapamycin, Ergtoxin had no effect on pluripotency markers (Fig. 8d), indicating that mTOR is downstream of membrane depolarization in hESCs as in *Xenopus*. Although rapamycin did reduce the final cell number per well, it inhibited the effect of Ergtoxin on pluripotency marker expression independently of the density at which cells were seeded (Supplementary Fig. 8e) and of the final cell number in the well (Supplementary Fig. 8f). Taken together, our data support that the polarization of membrane potential via KCNH channels promotes the exit from pluripotency and the activation of differentiated cell fates in *Xenopus* and human cells.

## Discussion

We propose a model in which membrane voltage regulates intracellular calcium during a critical stage of embryonic development, at which point cells need to extinguish pluripotency factors in order to activate a program of cellular differentiation (Fig. 8e). The K^+^ and Ca^2+^ channel network upstream of pluripotency factor expression potentially represents an extremely robust control mechanism over the first stages of organism development: ion channels are effective at low expression levels, modular and thus partially redundant with respect to each other (i.e., subunits of different channels can heterodimerize to form a channel if subunits of the same channel are unavailable), and their function depends on the existence of a simple ion concentration gradient across the plasma membrane. The basic elements of this regulation, K^+^ and Ca^2+^, are readily available extracellularly, conferring this system with some independence from protein-dependent cell signaling. Ultimately, ion channel networks may represent an additional mechanism to regulate cell fate during development, which is complementary to the established paradigm of gene expression regulation by secreted factors and ligand-receptor signaling (Fig. 8e).

Our work and the work by others supports the notion that membrane potential modulates development in a variety of contexts including LR patterning^2–6^, chondrocyte differentiation in chick limbs^13^ as well as morphogenesis of the developing Drosophila wing^8,9^. Additionally, our work adds to the molecular mechanisms that have associated membrane potential with stem cells differentation^12,14,60–62^. Of note, previous work has suggested that K^+^ itself may have signaling properties that affect the directed differentiation of hESCs^63^. However, our study indicates that it is V_m_ and not K^+^ itself that is critical. This is consistent with the notion that, because K^+^ is so abundant inside the cell, it is unlikely to act as a signaling moiety, unlike Ca^2+^.

While elevations in intracellular calcium do have physiological functions in the prospective mesoderm and ectoderm of the gastrula, both in patterning^24,25^ and morphogenesis^30^, we show that regulation of voltage-gated calcium channels by V_m_ is critical for the exit from pluripotency. Based on this, we suggest that low intracellular calcium reduces the expression of mTOR and pluripotency factors, which is conducive to differentiation onset. While we have not eliminated other voltage responsive signaling, our results indicate that calcium is critical in this context.

Regulation by V_m_ and Ca^2+^ presents interesting implications for developmental timing, as they appear to be key in specifying the exact time frame of pluripotency exit. Indeed, previous work has also implicated mTOR in developmental timing of the pluripotent state^64^, and our work demonstrates the upstream regulation of mTOR in this context. The requirement of V_m_ for this developmental period is also underscored by measurements in developing *Xenopus*^16^ and *Triturus*^15^ showing a clear hyperpolarization of V_m_ towards the onset of germ-layer differentiation that is concurrent with a reduction in calcium levels^26^. Therefore, it may be that membrane polarization may act as a timing mechanism to allow embryonic cells to be synchronized before the onset of differentiation. To do so, the embryo employs ion channels, mTOR, and an array of pluripotency transcription factors. Then secreted factors can act on these pluripotent cells to activate different programs of differentiation across the embryo (Fig. 8e). Of note, tissues with abnormal ion homeostasis at gastrulation onset do have differentiation potential and may eventually differentiate at later stages, but the significant delay in exiting pluripotency dramatically affects gastrulation morphogenesis itself as well as LR patterning.

Importantly, genetic data from Htx/CHD patients identified the KCNH family and especially *KCNH6*. Recent work has identified ion channels as an interesting intersection between CHD and autism^65^. While ion channels in neuronal development are well established^14^, our work adds to the role of ion channels in early embryonic development that includes pluripotency, LR patterning, and congenital heart disease.

Finally, the induction and maintenance of pluripotency clearly depends on a set of transcription factors, and our work connects V_m_ and intracellular calcium as upstream of this pluripotency program. This finding may be applicable to multiple contexts in which stem cells play a role in embryonic development or adult tissue homeostasis. Our work demonstrates the importance of V_m_ in vivo during early embryonic development as well as in vitro in human stem cells. Importantly, this pathway is readily manipulated by a wide range of highly specific channel inhibitors or simple changes in extracellular ionic concentrations. Therefore, we define multiple tools for pluripotency manipulations in embryos, organoids, and adult tissues where stem cells play a critical role.

## Methods

### *Xenopus* husbandry

In this study, we used *Xenopus tropicalis* from the N (Nigerian) strain. Adult *X. tropicalis* were raised and housed according to our established protocols which were approved by the Yale Institutional Animal Care and Use Committee. We induced ovulation, performed IVF, and raised embryos in 1/9× MR as previously described^66^. We staged *X. tropicalis* embryos according to Nieuwkoop and Faber^67^. In *Xenopus*, sex is determined in a 50:50 ratio genetically, and our experiments were all performed in embryos under 72 h of age before the sex of the animal can be determined morphologically.

### Morpholino oligonucleotides, mRNA, and CRISPRs

All injections of *Xenopus* embryos were performed at the one-cell stage using a fine glass needle and Picospritzer system, as previously described^68^. A *kcnh6* translation blocking (*kcnh6* MO, 5’-GGTCCTCGAAGTTTAGGATAAACAT-3’) and a scrambled morpholino oligonucleotide were obtained from Gene Tools LLC and injected at 10 ng to deplete *kcnh6* or as a control, respectively. CRISPR sgRNAs for *kcnh6* targeted either exon 3 or exon 4 based on the v7.1 gene model of the *X. tropicalis* genome (CRex3: 5’-GGAATAAGGGGTGAAGACAGCGG-3’ and CRex4: 5’-AGGGCGCTCTACATTTCCAATGG-3’). CRISPR sgRNAs for *cacna1c* (5’-GCAGACGGGGGCAGCGCCATTGG-3’) and *cacna1g* (5’-GGTTAATGGCTCTCAGCGGGCGG-3’) were designed from the v7.1 model of the *Xenopus tropicalis* genome. For F0 CRISPR knockdown, embryos were injected with 1.5 ng Cas9 Protein (PNA-Bio) and 400 pg of targeting sgRNA and raised to desired stages as previously described^69^. For *pitx2* and *coco* expression analyses, the dose of *kcnh6* sgRNA was halved to a subphenotypic dose of 200 pg to obtain embryos without gross morphological gastrulation defects. Full-length human *KCNH6* (NM_030779.3 cloned in pCS107), *GCaMP6* (subcloned in pCSDest) and *mCherry* cDNAs (Addgene #34935; in pCS2 + ), were used to generate capped mRNAs in vitro by first linearizing with appropriate restriction enzymes and then transcribing with the mMessage machine kit (Ambion). mRNAs were injected at 3 pg (human *KCNH6*), 150 pg (*GCaMP6*), and 150 pg (*mCherry*) per embryo. Embryos were raised at 21 °C to allow time for sufficient expression levels at blastula/gastrula stages.

### Inference of CRISPR edits (ICE) analysis

Genomic DNA from CRISPR and control embryos were obtained by lysing individual, stage 45 tadpoles in 50 mM NaOH as previously described^69^ and amplifying PCR fragments around the CRISPR target site that encompass ~200 bp upstream and 500 bp downstream of the site. The following primers were used for CRISPRs targeting exons 3 and 4 of the *kcnh6* locus, respectively: CRex3-F: 5’-CAGGACTGATGAAAGCAAGC-3’ and CRex3-R: 5’-GCTTATCCATAGCTGTAACAACG-3’; CRex4-F: 5’-GAGACAGTAGGCTGTTCC-3’ and CRex4-R: 5’-CCACAAGCAGTTTCACTACC-3’. PCR fragments were Sanger sequenced using the same forward primers, and sequencing traces were uploaded for analysis with the Synthego ICE analysis web tool to assess editing outcomes.

### Organ *situs*

Stage 45 *Xenopus* embryos were paralyzed with benzocaine or tricaine and scored with a light stereomicroscope. Cardiac looping was determined by position of the outflow tract; D-loop: rightward, L-loop: leftward; A-loop: midline. Normal intestinal looping was scored as counter-clockwise rotation of the gut, while abnormal intestinal looping was scored as completely inverse gut rotation (clockwise) or complete lack of looping (unlooped). While a completely inverted gut rotation is clearly an abnormality of LR patterning, an unlooped gut is less clear so we only considered an unlooped gut as abnormal *situs* when combined with abnormal placement (left-sided or midline) of the gall bladder. To quantify total abnormal organ *situs*, each tadpole was counted only once, regardless of whether multiple organs were affected.

### Whole mount in situ hybridization

Digoxigenin-labeled antisense probes for *pitx2* (TNeu083k20), *dand5/coco* (TEgg007d24), *myoD* (Tneu017H11), *myf5* (TGas127b01), *tbxt* (TNeu024F07), *foxj1* (Tneu058M03), *ectodermin* (TNeu104j16), *foxI1a* (Tgas002H16), *mixer* (TGas105b05), *vegT* (TGas066f22), *gsc* (TNeu077f20), *xnr3* (Tgas011k18), *vent2* (BG885317), *oct25* (TGas051h05), *oct60* (IMAGE: 7526158), *oct91* (IMAGE: 7575764), *vent1* (BG487195), *sox2* (Tgas061h22), *cytokeratin* (IMAGE:6991625), and *sox17β* (BG886038) were in vitro transcribed using T7 High Yield RNA Synthesis Kit (E2040S) from New England Biolabs. In order to generate a full-length antisense probe for *X. tropicalis kcnh6*, *kcnh6* cDNA was cloned from stage 45 tadpole whole mRNA using primers xtkcnh6-F: 5’- ATGTTTATCCTAAACTTCGAGGACC-3’ and xtkcnh6-R: 5’-CTAACTTCCTGGAAGACCTGGG-3’ (XM_012952904.1). We note that *kcnh6* had been misannotated as *kcnh2* in the v7.1 model of the *X. tropicalis* genome (We used NCBI Annotation XP_012808358.2 to identify KCNH6). Embryos were collected at the desired stages, fixed in MEMFA for 1–2 h at room temperature (RT) and dehydrated in 100% ethanol. GRPs were dissected post fixation and prior to dehydration to detect *dand5*. To detect putative gene expression in the prospective endoderm (*mixer*, *vegT*, *kcnh6*) gastrula stage embryos were bisected to facilitate better probe access. Briefly, whole mount in situ hybridization of digoxigenin-labeled antisense probes was performed overnight, the labeled embryos were then washed, incubated with anti-digoxigenin-AP Fab fragments (Roche 11093274910), and signal was detected using BM-purple (Roche 11442074001), as previously described in detail^68^.

### Medium conditions and treatments of embryos

Normal embryonic medium is 1/9x modified Ringer’s (MR) containing 11 mM NaCl, 0.2 mM KCl, 0.2 mM CaCl_2_, 0.1 mM Mg_2_Cl and 0.55 mM HEPES. To allow Ergtoxin to penetrate the embryos, we manually removed the vitelline envelope of stage 8 embryos and incubated embryos in 1/9× MR containing 50 nM Ergtoxin (Alomone STE-450) until stage 12. Embryos were then transferred back into 1/9× MR lacking Ergtoxin to develop until stage 45 in order to score organ *situs*. Barium chloride was applied into the medium at 20 mM and embryos were thoroughly rinsed in 1/9× MR after each incubation period for further development in Ba^2+^-free medium. For extracellular K^+^ manipulations, the KCl concentration in 1/9x MR was modified from 0.2 mM (normal) to 20 mM (high) or 0 mM (low). The ionophore valinomycin (ACROS) and L-type VGCC blocker Nifedipine (ACROS) were diluted in DMSO as stock solutions and applied to embryos in 1/9× MR at 2 and 10 μM, respectively. Treatments performed during gastrulation were applied from stage 8 through stage 12, and embryos were then rinsed thoroughly and returned into 1/9× MR. For rapamycin, we created a standard stock solution of 50 mg/ml in DMSO. The stock solution was diluted 1:2500 in the appropriate embryonic media (final 20 μg/ml). Embryos were treated at stage 7 and then fixed at stage 11 for in situ hybridization.

### GRP immunofluorescence

Embryos were fixed at stage 17 in 4% paraformaldehyde-PBS for 2 h at RT, washed in PBS, and then dissected to obtain GRPs. GRPs were permeabilized for 30 min at RT using 0.1% Triton-PBS (PBST), then blocked in 1% BSA-PBST for 1 h at RT and incubated in primary antibodies diluted in 1% BSA-PBST overnight at 4 °C (anti-myoD 1:100; LsBio C143580-100 or anti-acetylated tubulin 1:2000 Sigma T-6793). GRPs were then washed in PBST for 30 min and then incubated with secondary antibodies in 1% BSA-PBST for 1 h at RT. Phalloidin (1:50; Molecular Probes) and Hoechst 33342 (1:1000; Molecular Probes) were diluted into the secondary antibody solution. Images were acquired using a ZEISS 710 laser scanning confocal microscope.

### Intracellular V_m_ recordings

For recordings, devitellinized, stage 10 *kcnh6* or control MO-injected embryos were mounted into non-toxic clay with their animal pole exposed and covered with 1/9x MR. To investigate the resting potential, animal pole cells were impaled with a high-impedance (~70 MΩ), sharp microelectrode filled with 3 M KCl for intracellular recordings. The recordings were made using an Axon 200B amplifier and digitized using a Digidata 1320 digitizer. Jclamp software for Windows was used in current clamp mode. All electrodes were zeroed just before entry into the cells.

For the series of intracellular recordings in high K^+^ and choline treated embryos, stage 8–9 embryos were impaled similarly with an electrode of ~40 MΩ. These recordings were made using a HEKA EPC10 amplifier. We used HEKA PatchMaster v2x67software for Windows. All electrodes were zeroed just before entry into the cells.

### Calcium imaging

GCaMP6 and mCherry mRNAs were mixed and injected into embryos at the one-cell stage. Half of these embryos were then injected with *kcnh6* MO and the other half with control MO, still at the one-cell stage. Embryos were transferred at stage 10 into the round wells of a press-to-seal silicone isolator (Sigma S3685) mounted between two cover slips in 2% Methylcellulose-1/9x MR. GcaMP6 and mCherry fluorescence was then captured for 20 s (1 frame per second) via time lapse in the whole animal pole of each embryo with a ×20 objective of an LSM710 confocal microscope using identical acquisition settings across Control MO and *kcnh6* MO embryos. Time lapse recordings were conducted randomly and in an unbiased manner in regard to presence and intensity of calcium transients. However, all embryos did display transient increases in GcaMP6 fluorescence, varying in intensity and spreading to multiple cells. The frames of each recording were sorted to identify the calcium transient peak (in area), and GcAMP6 fluorescence intensity was quantified at peak as a ratio to mCherry in mCherry+ cells. The maximum Ca^2+^ transient area was calculated by demarcating in Fuji the GCaMP6(+) vs GcaMP6(–) area of the animal pole at transient peak. To avoid mosaicism artifacts, only embryos with even, non-mosaic mCherry expression across the entire animal pole were considered. To avoid embryonic stage dependent fluctuations in Ca^2+^ transient size, we verified each embryo for stage by progression of blastopore closure and alternated recordings of control and *kcnh6* MO embryos. Of note, there were no notable differences in mCherry expression between Control MO and *kcnh6* MO embryos.

### Animal cap pluripotency assays

After manually removing the vitelline envelope of stage 9 or 12 embryos, animal caps were excised and placed on agarose coated dishes in 1/9× MR solution. Caps were then directly placed into agarose coated wells of a 96-well plate in 1/3× MR containing 0.1% BSA and cultured without activin to allow for differentiation into epidermis, with low (20 ng/ml) activin to induce mesoderm, or high activin (200 ng/ml) to induce endoderm, as previously described^43^. Explants were raised at 25 °C until reaching the equivalent of stage 18 (monitored in whole embryos of the same batch), then fixed in 4% paraformaldehyde, washed in PBS, bleached to eliminate pigmentation (0.5× SSC, 5% formamide, 1.2% H_2_O_2_), and then processed by in situ hybridization as described above.

### RNA-seq

For RNA-Seq, embryos were kept at 25 °C either in 1/9× MR or in 10 mM KCl solution, and 10 embryos were harvested per time point and condition every 30 min starting at stage 8 and concluding at stage 13. Samples were immediately frozen and kept at −80 °C until homogenized in 100 μl Trizol spiked with ERCC RNA Spike-In Mix. 10 μl ERCC RNA Spike-In Mix (Thermo Fisher Scientific) were first diluted into a final volume of 870 μl DEPC water and then further diluted 1:10 into Trizol, which was used to homogenize the samples. Total RNA was purified from the embryo Trizol homogenates according to the manufacturer’s recommendations. After isopropanol precipitation, RNAs were resuspended in DEPC water and any contaminating genomic DNA was removed by overnight precipitation in 5 M LiCl at 4 °C. RNA was subsequently pelleted and washed twice with 70% ethanol. All RNAs were resuspended in DEPC water (2 μl/embryo), and finally, RNA quality was verified by Bioanalyzer. All libraries were sequenced with 100 bp paired-ends on an Illumina NovaSeq6000.

### Quantification RNA-seq

Stranded paired-end 100 bp RNA-seq reads were aligned to the Xt9.1 genome combined with ERCC spikes using STAR^70^ and quantified as transcripts per million (TPM) for each isoform with RSEM^71^ using the RSEM-STAR pipeline, with additional options “--seed 1618 --calc-pme --calc-ci --estimate-rspd --paired-end”. Using the ERCC spikes we identified a batch-dependent GC bias where AT-rich transcripts were preferentially lost as compared to GC-rich transcripts (Supplementary Fig. 7b). We leveraged knowledge of spike-in concentrations to build a GC model correction based on the dinucleotide content of RNAs. We calculate the propensity of each of the 16 dinucleotides (*AA, AC, …, TG, TT*) within each spike sequence, with *f*_*ik*_ is the frequency of dinucleotide *k* within sequence *i*, its propensity is ${p}_{{ik}}={f}_{{ik}}/{\sum }_{j}{f}_{{ij}}$. We then employ the following linear model to correct the TPM *t*_*si*_ of RNA spike ${i}$ in sample ${s}$ to its known concentration ${c}_{i}$:

$${{\log }}\,{c}_{i}={{\alpha }}+{{{\beta }}}_{{{T}}}{{\log }}\,{t}_{{si}}+{\sum }_{j}{\beta }_{j}{{\log }}\,{\rho }_{{ij}}$$

We use the GLM.jl (https://github.com/JuliaStats/GLM.jl) in the Julia language to apply this model and add a pseudocount of 2 to all dinucleotide frequencies. As the GC effect varies between UC and high K+ samples (Supplementary Fig. 7b), we apply the correction independently to UC and High K+. The correction is able to explain a significant proportion of variance in spike TPM, increasing R^2^ from 0.807 and 0.733 to 0.965 and 0.964 respectively from UC and high K+ samples. We apply this correction to each isoform of all genes quantified with the dinucleotide propensities of each isoform and RSEM isoform quantifications. We then sum all corrected quantifications at the isoform level to derive gene level quantifications. This allows us to account for differing isoforms of the same gene with differing dinucleotide propensities.

### Filtering of genes for differential expression analysis

We first filtered 34,192 quantified genes to find those with sufficient temporal expression for further analysis, we selected genes that had runs of 6 consecutive samples with uncorrected TPM > 0.4. This resulted 13,310 from which we excluded a further 162 genes which where excessively altered by the above described correction procedure, these had log2 fold changes between corrected or uncorrected quantifications outside of the interval (−2.5, 4.5). After dinucleotide correction and filtering we found excellent concordance between samples, with minimal evidence of outlying samples, by Spearman Correlation comparisons and principal components analysis (PCA) (Supplementary Fig. 7c, d). The two domains in visible in pairwise Spearman comparisons (Supplementary Fig. 7c) reflect the loss of maternal RNA and the commencement of widespread zygotic transcription as we previously described^19^. Projection onto the first two principal components revealed that samples lie in appropriate order on a trajectory in 2D space, and the largest divergences between UC and high K+ occur midway through the time series in agreement with Gaussian process differential expression and clustering described below. Corrected dinucleotide gene expression abundances are used in all analyses.

### Temporal differentiation expression

To determine genes temporally differentially expressed we used Gaussian process (GP) regression as we have previously applied^19^. All GP regression was performed with GaussianProcesses.jl (https://github.com/STOR-i/GaussianProcesses.jl; https://arxiv.org/abs/1812.09064). Due to the overdispersed nature of RNA-seq count data, we apply a variance stabilizing transform that puts all genes on the same scale: ${y}_{{si}}=\sqrt{\alpha+\beta {x}_{{si}}/{m}_{i}}$, with *x*_*si*_ the dinucleotide corrected abundance of gene *i* in sample *s*, *m*_*i*_ the maximum *x*_*si*_ over all samples, and *α* = 1, *β* = 1000. We then perform exact GP regression (GP prior and a Gaussian likelihood) with Matern52 kernel, we optimize the three associated hyperparameters: ${\sigma }_{f}^{2}$ the signal variance, *τ* the timescale (using previous terminology^19^, this parameter is commonly referred to as the lengthscale *l*), and ${\sigma }_{n}^{2}$ the sample noise variance. Parameters are selected by optimizing marginal log-likelihood with parameters in log space: ${{\log }}{\sigma }_{f},{{\log }}\tau,{{\log }}{\sigma }_{n}$, and to ensure physiologically reasonable values for each we place Gaussian priors, ${{{{{\mathscr{N}}}}}}\left(\mu,\sigma \right)$ over each of these variables respectively ${{{{{\mathscr{N}}}}}}\left(1.4,4.0\right){{{{{\mathscr{,}}}}}}{{{{{\mathscr{N}}}}}}\left(1.2,1.0\right){{{{{\mathscr{,}}}}}}{{{{{\mathscr{N}}}}}}(1.0,0.75)$. Finally, we reported GP median and 95% confidence intervals through our inverted data transformation ${\hat{x}}_{{si}}={m}_{i}({\hat{y}}_{{si}}^{2}-\alpha )/\beta$ and set ${\hat{x}}_{{si}}=0$ for ${\hat{y}}_{{si}} < \sqrt{\alpha }$.

To determine temporal differential expression, we calculate a marginal likelihood ratio for whether we prefer separate GP models for UC and high K+ or a single GP model for all data combined. If *L*_*U*_ and *L*_*K*_ are the marginal log-likelihoods for UC and high K+ respectively, and *L*_*UK*_ is the marginal log-likelihood for a single regression through UC and high K+ together. Then we calculate log-likelihood ratio ${LR}={L}_{U}+{L}_{K}-{L}_{{UK}}$ of evidence in favor of two models (essentially that the UC and High K+ have different expression trajectories for a given gene) and determine genes with *LR* > 0 as temporally differentially expressed. This resulted in 5144 differentially expressed genes, with 4043 activated and 1101 repressed (Supplementary Fig. 7d which shows that the max absolute divergence z-score between UC and high K+ trajectories increases with *LR*). We also considered a more stringent condition for differential expression using the Bayesian Information Criterion (BIC)^19^, that resulted in 2388 differentially genes. We found that this diminished differential expression gene set enrichments described below, indicating that the BIC was too conservative and we continued with our condition based on log-likelihood ratio.

### Clustering

To determine sets of differentially expressed genes with similar trajectories, we applied K-means clustering to activated and repressed genes independently. We define a gene as activated if Gaussian process median for High K+ exceeds UC on average, and repressed if it does not, we found no genes for which the mean of High+ and UC differences was zero. We cluster UC and High K+ genes by taking Gaussian process medians and normalizing by the maximum value experience by UC or High K+. We then cluster both trajectories together employing the kmeans function offered by Clustering.jl (https://github.com/JuliaStats/Clustering.jl) with default settings and random seed 16. To select the cluster number, we calculated the silhouette score for activated and repressed clusters for k = 2–10. We found that the maximal mean silhouette score activated genes was k = 3 and for repressed genes was k = 2, but that scores were broadly similar for k = 2–4 and decreased significantly for k > 4, suggesting that k = 4 provides a reasonable partition of the data. In line with this we explored the clusters from k = 2–10, and found that key clusters were not well-resolved for k < 4 and that k > 4 clusters refined k = 4 behaviors. As k > 4 did not reveal new behaviors and did not improve gene set enrichments, we selected k = 4 to cluster activated and repressed genes.

### Gene set enrichments

To assess the composition of each cluster we performed gene set enrichments using Enrichr^45^. We took genes from each cluster with a known *Xenopus* gene symbol and converted these to human symbols, by removing any “.N” suffix for an integer N (for example, ventx1.1 becomes ventx1) and converting to uppercase. We then made the following substitutions to convert certain known *Xenopus* gene symbols to human where the name of the ortholog has diverged or only a paralog exists: pou5f3 → POU5F1, mix1 → mixl1, dppa2 → DPPA4, lefty → lefty2, ventx1-3 → NANOG, mespb → MESP1, sox17a/b → SOX17. We remove any duplicate names that arose in this process. We calculated enrichments for the following gene sets: KEGG_2019_Human, BioPlanet_2019, WikiPathways_2019_Human, GO_Biological_Process_2018, GO_Molecular_Function_2018, GO_Cellular_Component_2018, ChEA_2016. We calculate enrichments for each cluster individually and consecutive combinations of the 4 clusters: 1, 2, 3, 4, 12, 23, 34, 123, 234, 1234. All enrichments can be found in Supplementary Data 1, selected enrichments are given in Fig. 6C and terms are given shortened labels for brevity (Table 2):

**Table 2:** Selected enrichments from RNA-seq time course

Label	Term	Gene set
mTOR	mTOR signaling pathway	KEGG_2019_Human
Autophagy	Autophagy	KEGG_2019_Human
Ubiquitin	Ubiquitin-protein transferase activity GO:0004842	GO_Molecular_Function_2018
ER	Protein processing in endoplasmic reticulum	KEGG_2019_Human
Spliceosome	Spliceosome	KEGG_2019_Human
WNT signaling	Wnt signaling pathway	KEGG_2019_Human
Pluripotency signaling	Signaling pathways regulating pluripotency of stem cells	KEGG_2019_Human
Endoderm differentiation	Endoderm differentiation WP2853	WikiPathways_2019_Human

Selected enrichments and their associated abbreviated label listed in Fig. 6c.

### Motif analysis

To find motifs enriched in the promoters, we took the 500 bp upstream of the promoter of the maximally expressed isoform for each gene in the four activated and four repressed clusters, along with a background of the 500 bp upstream of all annotated TSS in the Xt9.1 genome. We extracted fasta files for each of these sets of regions, and then used findMotifs.pl from Homer^72^ to search for known motifs with options: “findMotifs.pl cluster*AB*.fa fasta out*AB* –fasta background.fa –nomotif” where *A* ∈ {activated, repressed} and *B* ∈ {1, 2, 3, 4}. We filtered results to select best matching motifs from related families, namely we collapsed all ETS motifs to the canonical Homer ETS promoter motif; all SP and KLF motifs to SP1; SOX motifs to SOX2; all HOX motifs to HOXD13 (the highest scoring HOX); we represent all GFY and Ronin matches as ZNF143 (for which the motifs overlap); and we excluded motif annotated as PRDM10, due to low confidence in the motif. The motif annotated as ATF1 is an example of the cAMP response element (CRE) bound by CREB factors including ATF1, we label this as CRE/ATF1. The top 16 motif enrichments are given in Supplementary Fig. 7h, in Fig. 6d we give the top 6 motif matches, excluding ZNF143 due to divergence from the JASPAR database, to which we add CRE/ATF1 as a putative calcium responsive element motivated by CREM/CREB1 gene set enrichments Supplementary Fig. 7j).

To calculate CRE and ETS motif enrichment for mTOR and pluripotency genes, we took genes annotated with the terms *mTOR* signaling pathway and Signaling pathways regulating pluripotency of stem cells from KEGG_2019_Human as provided by Enrichr^45^ that are activated in high K+ (LR > 0) and are present in clusters A1 and A2. The resulting genes were subjected to the same promoter analysis, using Homer to calculate the occurrence of the maximal ATF/CRE family motif and the ETS motif in these promoters and the background set to report Fisher Exact test p-values and Odds Ratios.

### *Xenopus* biological replicates, statistical methods, graphs, and models

In experiments where embryos were evaluated for phenotypes and scored (gastrulation, left-right patterning, in situ hybridizations) we carried out three to five biological replicates and Fisher’s exact test to evaluate statistical significance. The animal cap experiment was performed twice with a total score of four to eight animal caps per experiment. For the calcium transient analyses, data was collected from three to five embryos in each experiment in three independent experiments, and statistical analyses on GCaMP/mCherry fluorescence intensity as well as Ca^2+^ transient area were performed using student’s *t*-test. For whole cell electrophysiological recordings, three to five embryos (two cells each) were examined for their membrane potential and statistical significance was tested by student’s *t*-test. Graphs were designed using GraphPad Prism software. Models were created with BioRender.com.

### hESC culture

hESCs were grown in mTeSR1 (STEMCELL Technologies) in tissue culture dishes coated with Matrigel (Corning; 1:200 in DMEM/F12) and kept at 37 °C, 5% CO_2_. The cell lines used were ESI017 (ESIBIO) and H9. Cells were routinely passaged using dispase (STEMCELL Technologies) and tested for mycoplasma contamination and found negative. For rapamycin experiments, cells were grown in MEF-conditioned HUESM media supplemented with 20 ng/ml bFGF as previously described^73^ with or without 100 nM rapamycin, which we found to increase the survival of rapamycin treated cells compared to cells grown in mTeSR1.

### hESC treatments and differentiation

Cells were dissociated with accutase and seeded onto eight-well imaging slides (ibidi 80826) at a density of 4–6 × 10^4^/cm^2^. Cells were seeded and maintained in Rock-inhibitor Y27672 (MCE; 10 μM) to increase survival and the uniformity of response. Treatments with 1 mM BaCl_2_ or 10 or 25 nM Ergtoxin or 100 nM rapamycin were initiated 4 h after seeding. Differentiation was initiated 24 h after seeding where indicated. To initiate differentiation, the media was replenished with/without BaCl_2_ or Ergtoxin and treated with the indicated growth factors or small molecules. The media with any treatments was replenished daily.

### Immunofluorescence of hESCs

Cells were fixed for 30 min in 4% paraformaldehyde, rinsed twice with DPBS (without Ca^2+^ and Mg^2+^, denoted DPBS-/-), and blocked for 30 min at room temperature. The blocking solution contained 3% donkey serum and 0.1% Triton X-100 in 1× DPBS^−/−^. After blocking, the cells were incubated with primary antibodies at room temperature for 2 hours. Antibodies and concentrations are listed in Supplementary Table 1. Cells were washed three times with DPBST (1X DPBS-/- with 0.1% Tween 20) and incubated with secondary antibodies (AlexaFluor 488 A21206, AlexaFluor 555 A31570 and A21432, and AlexaFluor 647 A31571, Thermo Fisher; 1:500) and DAPI for 30 min at room temperature. After secondary antibody incubation, samples were washed in DPBST and then DPBS at room temperature.

### hESC Imaging and analysis

Images were acquired using a ×20, NA 0.75 objective on an Olympus IX83 inverted epifluorescence microscope or an Olympus/Andor spinning disk confocal microscope. Cell segmentation was performed using ilastik software^74^. This segmentation was cleaned (to remove debris and to separate merged cells) and mean nuclear protein intensities as well as standard errors were quantified using a custom MATLAB code. Nuclear intensities were normalized by DAPI to correct for intensity variation due to optics. Code is available at https://github.com/warmflashlab/Sempou2022_Code.

### qPCR

For qPCR, hESCs were grown with or without ErgToxin (25 nM) for the indicated times. RNA collection and DNase treatment were performed using the RNAqueous®-Micro Total RNA Isolation Kit (AM1931) and cDNA was synthesized with the SuperScript Vilo cDNA Synthesis Kit (Fisher Scientific 11754-050). qPCR measurements were collected using SYBR Green reagent (LifeTech-4367659) on a Step OnePlus instrument (Applied Biosciences). Data were normalized using the housekeeping gene GAPDH. Primers for qPCR were:

*OCT4*: 5’-caagctcctgaagcagaagag−3’, 5’-ccaaacgaccatctgccgcttt−3’,

*SOX2*: 5’-ccatgcaggttgacaccgttg−3’, 5’-tcggcagactgattcaaataata-3’,

*NANOG*: 5’-tgggatttacaggcctgagcca−3’, 5’-aagcaaagcctcccaatcccaaa−3’,

GAPDH: 5’-caccgtcaaggctgagaacg-3’, 5’-gccccacttgattttggagg-3’.

### Statistics and reproducibility

Embryo and cell sample sizes in this study were chosen according to the standards in the field. No data were excluded from the analyses. The investigators were blinded to allocation during experiments and outcome assessment whenever feasible.

For statistics, two-sided Fisher’s exact test was used to assess significance of two variables with independent proportions, (treated/untreated) and their outcomes (normal/abnormal). This test was applied when scoring for morphological phenotypes of embryos and WMISH results, under control conditions or after microinjection (CR, MO, mRNA), medium manipulations or treatment with compounds. Two-tailed, unpaired *t*-tests or ANOVA were used to test whether the means of two populations differ largely from one another and were employed for V_m_, calcium intensity and calcium transient area comparisons as well as all hESC experiments.

### Reporting summary

Further information on research design is available in the Nature Research Reporting Summary linked to this article.

## Supplementary information

Supplementary Information

Peer Review File

Description of Additional Supplementary Files

Supplementary Data 1

Supplementary Movie 1

Supplementary Movie 2

Reporting Summary

## Data Availability

The RNA-seq time series of uninjected-control (UC) and high K+ embryos generated in this study have been deposited in the Gene Expression Omnibus under accession GSE186670. Source data are provided with this paper.

## Source data

Source Data

## Code availability

All source data and code required to analyze RNA-seq time series and generate figures are available at https://github.com/owensnick/KCNH6GenomicsFigures.jl, 10.5281/zenodo.6967350. Source code for quantitating protein intensities in human ES cells is available at https://github.com/warmflashlab/Sempou2022_Code.
