# Author Correction: Membrane potential drives the exit from pluripotency and cell fate commitment via calcium and mTOR

**DOI:** 10.1038/s41467-023-39025-z

**Published:** 2023-06-05

**Authors:** Emily Sempou, Valentyna Kostiuk, Jie Zhu, M. Cecilia Guerra, Leonid Tyan, Woong Hwang, Elena Camacho-Aguilar, Michael J. Caplan, David Zenisek, Aryeh Warmflash, Nick D. L. Owens, Mustafa K. Khokha

**Affiliations:** 1 Pediatric Genomics Discovery Program, Departments of Pediatrics and Genetics, Yale University School of Medicine, 333 Cedar Street, New Haven, CT 06510 USA; 2 Department of Cellular and Molecular Physiology, Yale University School of Medicine, 333 Cedar Street, New Haven, CT 06510 USA; 3 Departments of Biosciences and Bioengineering Rice University, 345 Anderson Biological Labs, Houston, TX 77005 USA; 4 Department of Clinical and Biomedical Sciences, University of Exeter, Barrack Road, Exeter, EX2 5DW UK

**Keywords:** Pluripotency, Embryology, Mesoderm, Ion channel signalling

Correction to: *Nature Communications* 10.1038/s41467-022-34363-w, published online 05 November 2022

The original version of this Article contained an error in Fig. 2i, in which the left y-axis was labelled ‘2.5 x 10^3^, 1.0 x 10^3^, 1.0 x 10^3^, 1.0 x 10^4^, 1.25 x 10^4^’. It should have been labelled 2.3 x 10^3^, 5.0 x 10^3^, 7.5 x 10^3^, 1.0 x 10^4^, 1.25 x 10^4^’. This has been corrected in the PDF and HTML versions of the Article.

