# Supplementary material for: Membrane potential drives the exit from pluripotency and cell fate commitment via calcium and mTOR
Source: Nat Commun. 2022 Nov 5;13:6681. doi: 10.1038/s41467-022-34363-w (PMC9637099; doi:10.1038/s41467-022-34363-w)
Supplement: Supplementary file 1 — Supplementary Information [file 41467_2022_34363_MOESM1_ESM.docx]

***Supplementary information for***

**Membrane potential drives the exit from pluripotency and cell fate commitment via calcium and mTOR**

**Emily Sempou^1^, Valentyna Kostiuk^1^, Jie Zhu^2^, M. Cecilia Guerra^3^, Leonid Tyan^1,2^, Woong Hwang^1^, Elena Camacho Aguilar^3^, Michael J. Caplan^2^, David Zenisek^2^, Aryeh Warmflash^3^, Nick D. L. Owens^4^, Mustafa K. Khokha^1^**

### ^1^Pediatric Genomics Discovery Program

### Departments of Pediatrics and Genetics

### Yale University School of Medicine,

### 333 Cedar Street

### New Haven, Connecticut 06510, USA

^2^Department of Cellular and Molecular Physiology

### Yale University School of Medicine

### 333 Cedar Street

### New Haven, Connecticut 06510, USA

^3^Departments of Biosciences and Bioengineering
Rice University

345 Anderson Biological Labs

Houston, Texas 77005, USA

^4^Department of Clinical and Biomedical Sciences**,**

**University of Exeter,**

**Barrack Road, Exeter, EX2 5DW, United Kingdom**

*Correspondence: mustafa.khokha@yale.edu

**
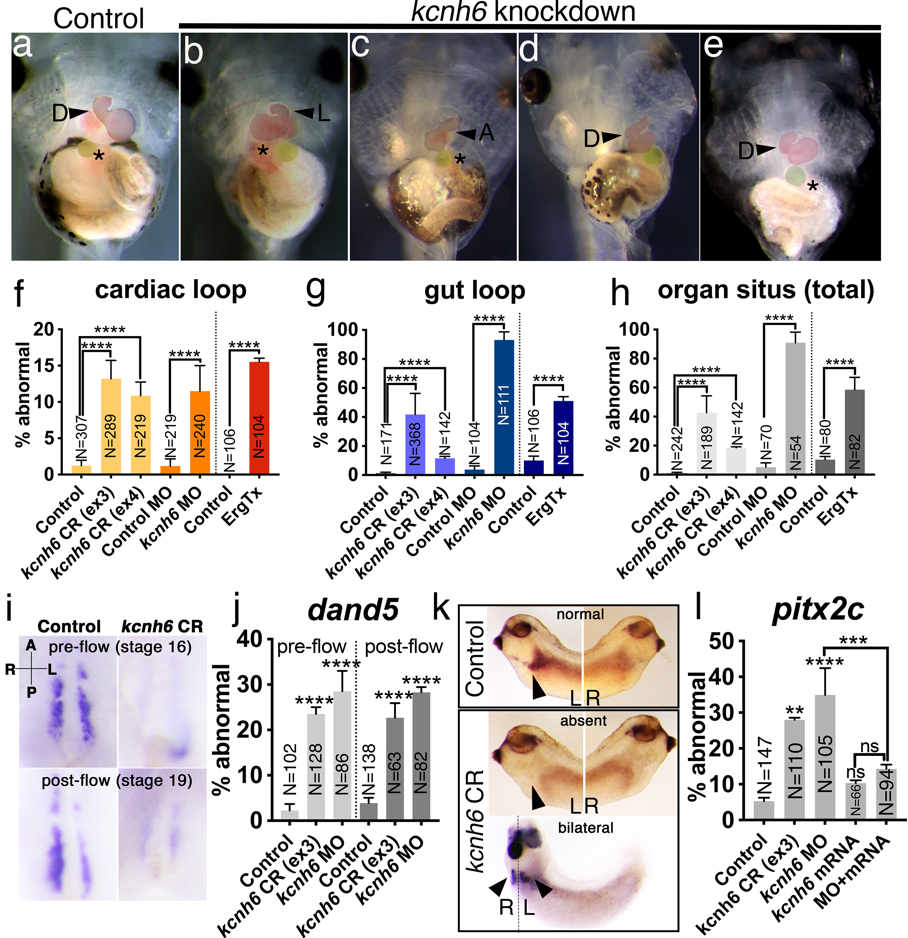
**

**Supplementary Figure 1.** ***kcnh6* depletion or Ergtoxin induces LR patterning defects**.

**a-e)** Examples of organ *situs* in stage 47 *X. tropicalis* tadpoles (ventral view with anterior to the top). Arrowheads indicate normal (D) and abnormal (L and A; A=outflow tract is midline) cardiac looping; the heart and gall bladder are minimally pseudo-colored in pink and green respectively for visualization; asterisks indicate the location of the liver whenever discernible.

**f-h)** Percentages of stage 47 tadpoles with *situs* defects upon injection of CRISPRs targeting independently two sites in different exons of the *kcnh6* locus, translation blocking MO, or after treatment with Ergtoxin. Graphs report mean ±SEM. p-values for (f) are (*kcnh6*CRex3 vs Co)=6.02e-008, (*kcnh6*CRex4 vs Co)=4.47e-006, (*kcnh6*MO vs ControlMO)=4.57e-005, (ErgTx vs Co)=1.08e-007; p-values for (g) are (*kcnh6*CRex3 vs Co)=5.64e-012, (*kcnh6*CRex4 vs Co)=8.39e-007, (*kcnh6*MO vs ControlMO)=6.06e-046, (ErgTx vs Co)=8.14e-012; p-values for (h) are (*kcnh6*CRex3 vs Co)=3.08e-024, (*kcnh6*CRex4 vs Co)=2.17e-010, (*kcnh6*MO vs ControlMO)=5.70e-025, (ErgTx vs Co)=1.54e-015; two-sided Fisher’s exact test. Total tadpole numbers (N) in graphs were collected over at least 3 independent experiments; Ergtoxin data is from 2 independent experiments; Tadpoles with multiple defects were counted once.

**i-j)** Detection of *dand5* expression via WMISH in stage 16 (pre-ciliary flow) and stage 19 (post flow) embryos viewed ventrally (i; A= anterior, P= posterior; R= right, L= left); (j) indicates percentages of embryos with absent *dand5* expression.

**k-l)** Detection of *pitx2c* expression in stage 28 embryos via WMISH; embryos in (k) are lateral views with dorsal to the top and either the left (L) or right (R) side visible; (l) indicates percentages of embryos with abnormal (absent or bilateral) *pitx2c* expression.

Graphs (j) and (l) report mean ±SEM. Total tadpole numbers (N) in graphs were collected over at least 2 independent experiments; p-values in (j) are (*kcnh6*CRex3 vs Co, pre-flow)=8.22e-008, (*kcnh6*MO vs Co, pre-flow)=1.50e-008, and (*kcnh6*CRex3 vs Co, post-flow)=8.34e-005, (*kcnh6*MO vs Co, post-flow)=2.68e-007; p-values in (l) are (*kcnh6*CRex3 vs Co)=2.33e-003, (*kcnh6*MO vs Co)=1.55e-010, (MO+mRNA vs MO)=6.75e-004; two-sided Fisher’s exact test. Key for asterisks: *=p≤0.05, **=p≤0.01, ***=p≤0.001, ****=p≤0.0001; ns= non-significant for p>0.05.

Source data are provided as a Source Data file.


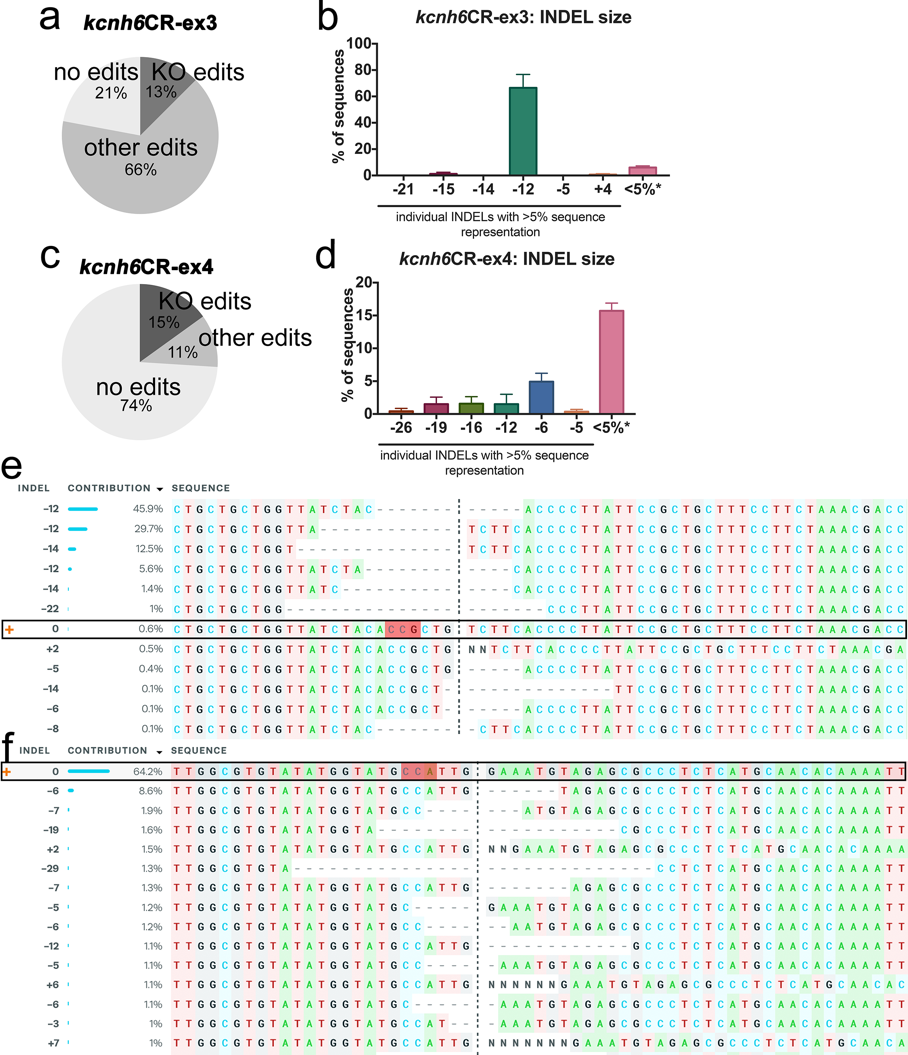


**Supplementary Figure 2. Inference of Crispr Edits (ICE) analysis in *kcnh6* CRISPR stage 47 tadpoles.**

**a,c)** Average percentages of frameshift (KO= knockout), in-frame (other) or unedited *kcnh6* (no edits) sequences for CRISPRs targeting exon 3 (a) or exon 4 (c).

**b,d)** Distribution of INDEL sizes; INDELs with representation >5% of total sequences are represented in individual bars, whereas those with <5% are summed in the last bar (*). Graphs display mean ±SEM; 16 tadpoles were analyzed for each CRISPR (control, ex3 or ex4) over 2 independent experiments.

**e,f)** Representative examples of CRISPR edits by (e) *kcnh6* CR exon 3 or (f) *kcnh6* CR exon 4. Sequences from two individual tadpoles are shown. The unedited control sequence is highlighted by a black box and the PAM sequence is in red; vertical dotted lines indicate the presumptive cut site.

Source data are provided as a Source Data file.

**
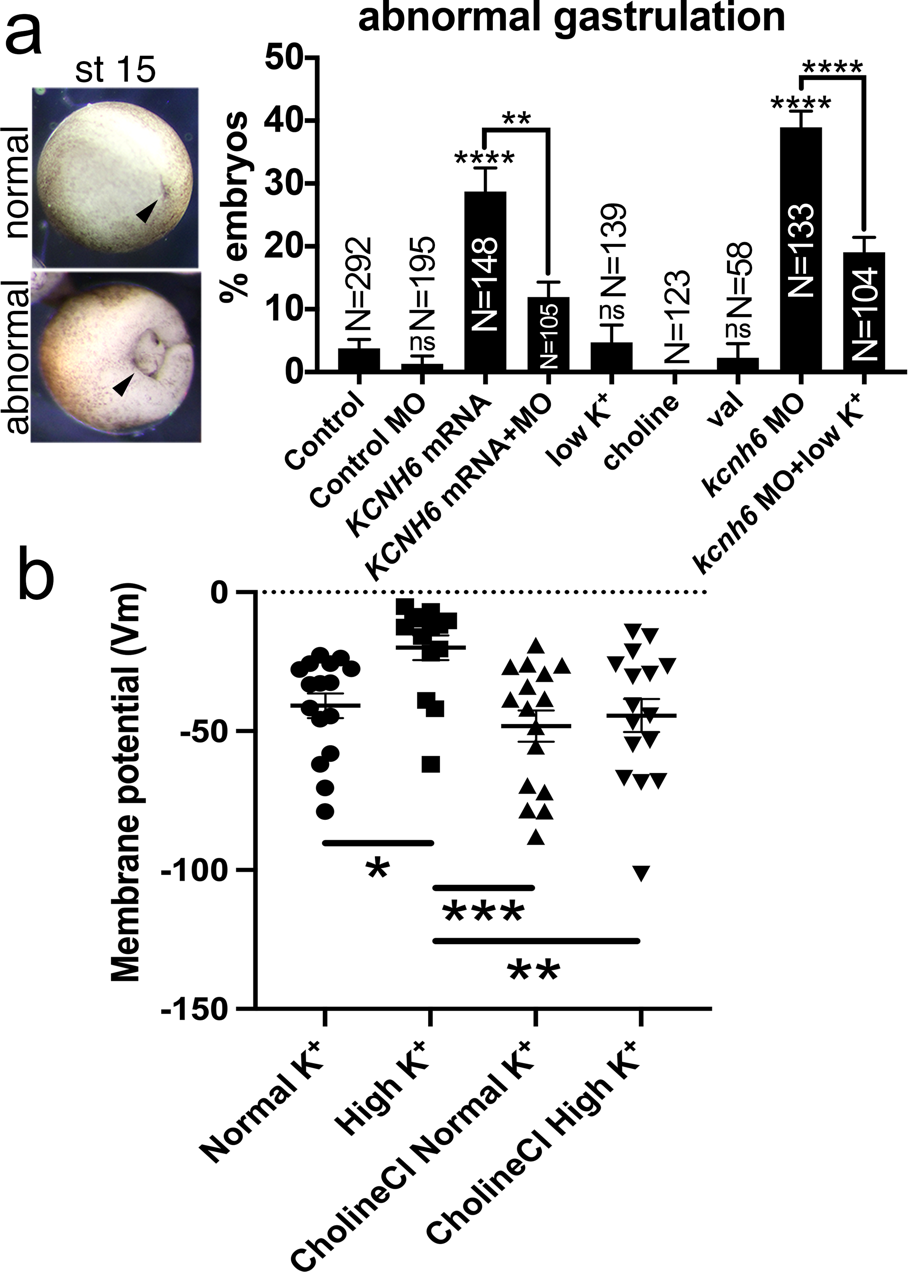
**

**Supplementary Figure 3. Controls to Figure 1m and V_m_ measurements of High K^+^ and choline embryos**

**a)** *E*ffects of KCNH6 mRNA expression and hyperpolarizing conditions on gastrulation in control or kcnh6 MO embryos. Percentages of embryos with incomplete blastopore closure at stage 17. Graph presents mean ±SEM; *p-values* are: (*kcnh6*mRNA vs Co)=4.22e-037, (*kcnh6*MO+mRNA vs mRNA)=4.00e-003, *(kcnh6*MO vs ControlMO*)=*2.87e-025, (*kcnh6* MO+lowK^+^ vs *kcnh6*MO)=8.91e-005; two-sided Fisher’s exact test. Total embryo numbers (N) are from at least 2 independent experiments. Key for asterisks: *=p≤0.05, **=p≤0.01, ***=p≤0.001, ****=p≤0.0001; ns= non-significant for p>0.05.

**b)** Intracellular recordings in the prospective ectoderm of stage 9 embryos; V_m_ was measured relative to the medium*.* *C*holineCl refers to substitution of ½ Na^+^ with choline chloride*.* Centers of bars represent mean, error bars represent ±SEM. Each data point represents one cell*.* Normal K*^+^* (n=16 cells*/*14 embryos) vs. High K^+^ (n=14 cells*/*13 embryos) p=0.04, High K^+^ vs. CholineCl Normal K*^+^* (n=16 cells*/*12 embryos) p=0.002, High K*^+^* vs. CholineCl High K^+^ (n=16 cells*/*12 embryos) p=0.01, one-way ANOVA with Bonferroni correction; Total embryo numbers collected over 3 independent experiments. Key for asterisks: *=p≤0.05, **=p≤0.01, ***=p≤0.005, ****=p≤0.0001.

Source data are provided as a Source Data file.

**
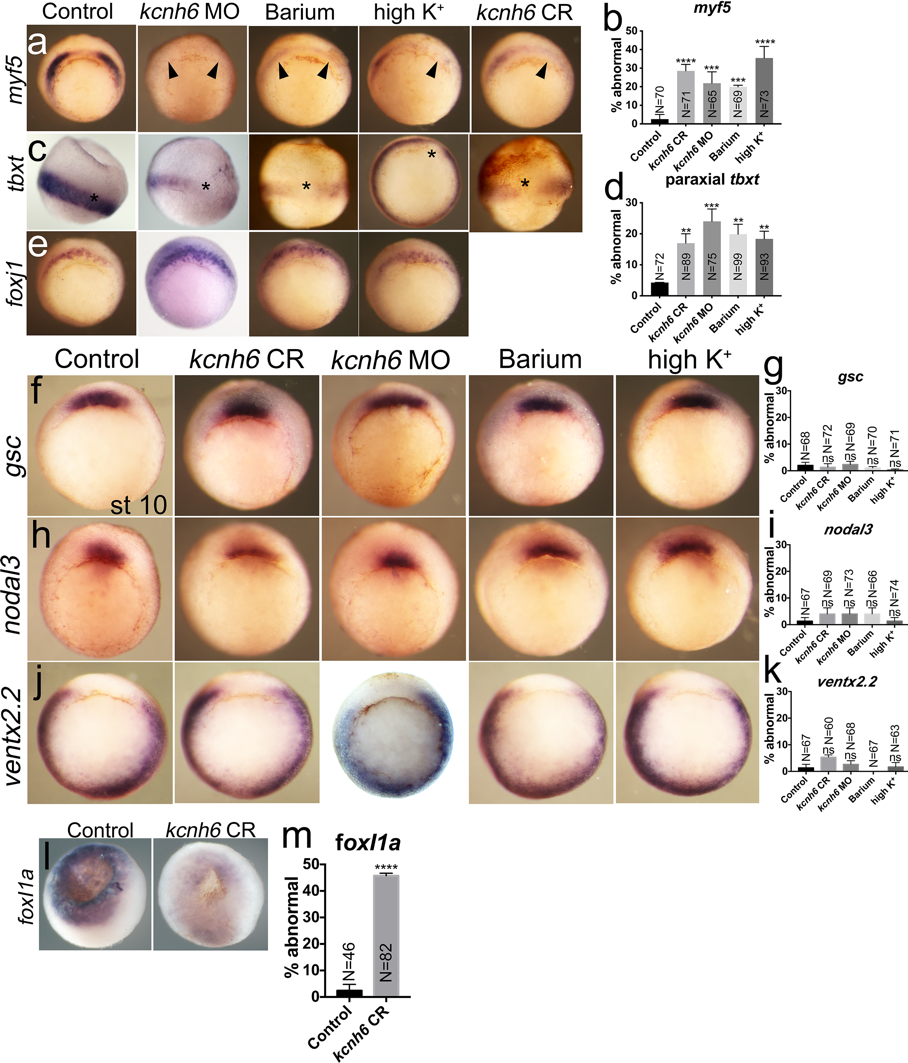
**

**Supplementary Fig 4. Effects of kcnh6 depletion and V_m_ depolarization on germ layer differentiation.**

**a-e)** WMISH for mesodermal transcripts in stage 10 embryos. a) Vegetal views with dorsal to the top showing expression of paraxial mesodermal marker *myf5*. b) Percentages of embryos with absent or reduced (abnormal) *myf5* expression; p-values are (*kcnh6*CR vs Co)=7.12e-005, (*kcnh6*MO vs Co)=4.44e-004, (Ba^2+^ vs Co)=3.03e-004, (highK^+^ vs Co)=7.38e-007; two-sided Fisher’s exact test. c) Lateral views showing *tbxt* expression (highK^+^ is a vegetal view); asterisk (*) marks absent expression in the paraxial region of the mesoderm. d) Percentages of embryos with abnormal *tbxt* expression (as shown in (c)); p-values are (*kcnh6*CR vs Co)=6.93e-003, (*kcnh6*MO vs Co)=6.77e-004, (Ba^2+^ vs Co)=4.61e-003, (highK^+^ vs Co)= 4.05e-003; two-sided Fisher’s exact test. (e) vegetal views with dorsal to the top showing *foxj1* expression, see Main Figure 4 for quantification.

**f-k)** WMISH for transcripts of dorsal organizer (*gsc*, *xnr3*) and ventral mesoderm (*ventx2.2*) marker genes; vegetal views of stage 10 embryos with dorsal to the top; (g, i, and k) show percentages of stage 10 embryos with abnormal expression of these markers (no effects are observed; non-significant, p>0.05; two-sided Fisher’s exact test).

**l,m)** WMISH for *foxI1a*, expressed in the prospective ectoderm; animal views. (m) shows percentages of embryos with absent *foxI1a* expression; p-value (*kcnh6*CR vs Co)=3.09e-006; two-sided Fisher’s exact test.

All graphs show mean ±SEM; total embryo numbers (N) in the graphs are from 3 independent experiments; data for *foxI1a* are from 2 independent experiments; key for asterisks: ***=p≤0.001, ****=p≤0.0001, ns=non-significant for p>0.05.

Source data are provided as a Source Data file.

**
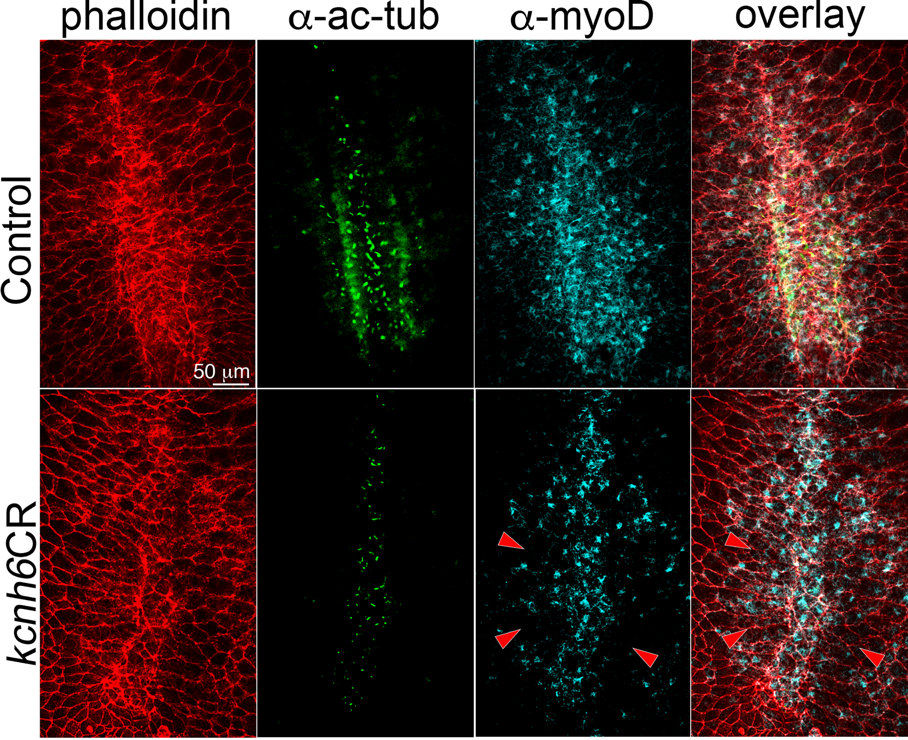
**

**Supplementary Figure 5. Effects of *kcnh6* depletion on LRO patterning.**

Immunostaining of the gastrocoel roof plate, where the LRO is located, in stage 17 embryos for acetylated tubulin (cilia) and MyoD (paraxial mesoderm); cell morphology is highlighted with phalloidin (actin); ventral views with anterior to the top; arrowheads indicate areas of absent MyoD labelling in the paraxial margins of the LRO in *kcnh6* CRISPR embryos. Representative images shown from a total of N=12 gastrocoel roof plates per condition, collected over 2 independent experiments.


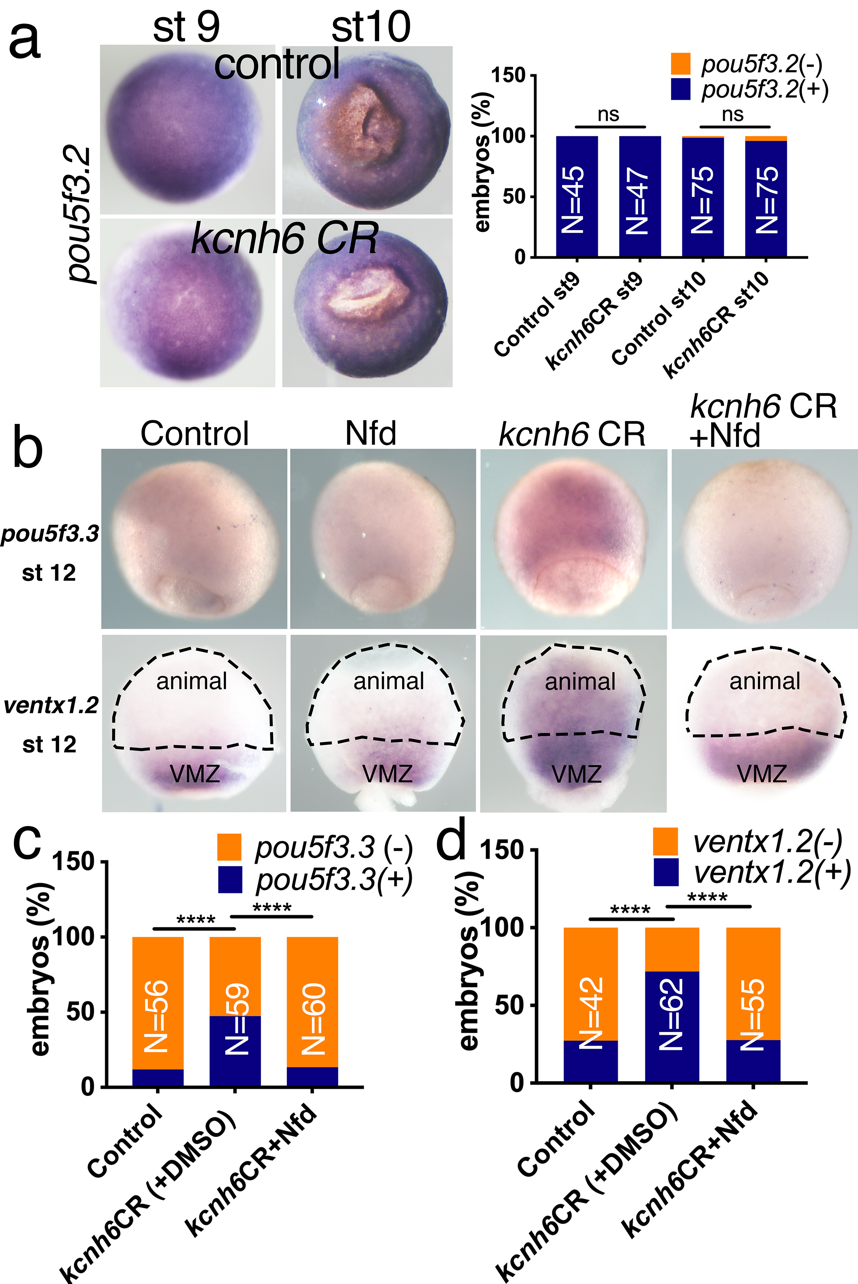


**Supplementary Figure 6. Pou5f3.2 and the role of VGCCs in pluripotency.**

**a)** animal pole view; WMISH detecting *pou5f3.2* expression in stage 10 control and *kcnh6* CR (CRISPR) embryos, quantitated in right panel. Graph shows mean percentages of embryos with absent(-)/present(+) *pou5f3.2* expression; total embryo numbers (N) in graph from 3 independent experiments; ns=non-significant for p>0.05 by two-sided Fisher’s exact test.

**b)** lateral views with animal pole to the top; WMISH for *pou5f3.3* and *ventx1.2* in stage 12 embryos, quantified in (c) and (d) respectively; Nfd (nifedipine), VMZ (ventral marginal zone).

**c and d)** Mean percentages of embryos with present/absent *pou5f3.3* (c) and *ventx1.2* (d) expression; p-values in (c) for *pou5f3.3* are (*kcnh6*CR+DMSO vs Control+DMSO)=4.54e-005, (*kcnh6*CR+Nfd vs *kcnh6*CR+DMSO)=5.60e-005 and in (d) for *ventx1.2* (*kcnh6*CR+DMSO vs Control+DMSO)= 2.14e-006, (*kcnh6*CR+Nfd vs *kcnh6*CR+DMSO)=2.87e-006; two-sided Fisher’s exact test; total embryo numbers (N) in graph are from 3 independent experiments. Key for asterisks: ****=p≤0.0001; ns= non-significant for p>0.05.

Source data are provided as a Source Data file.


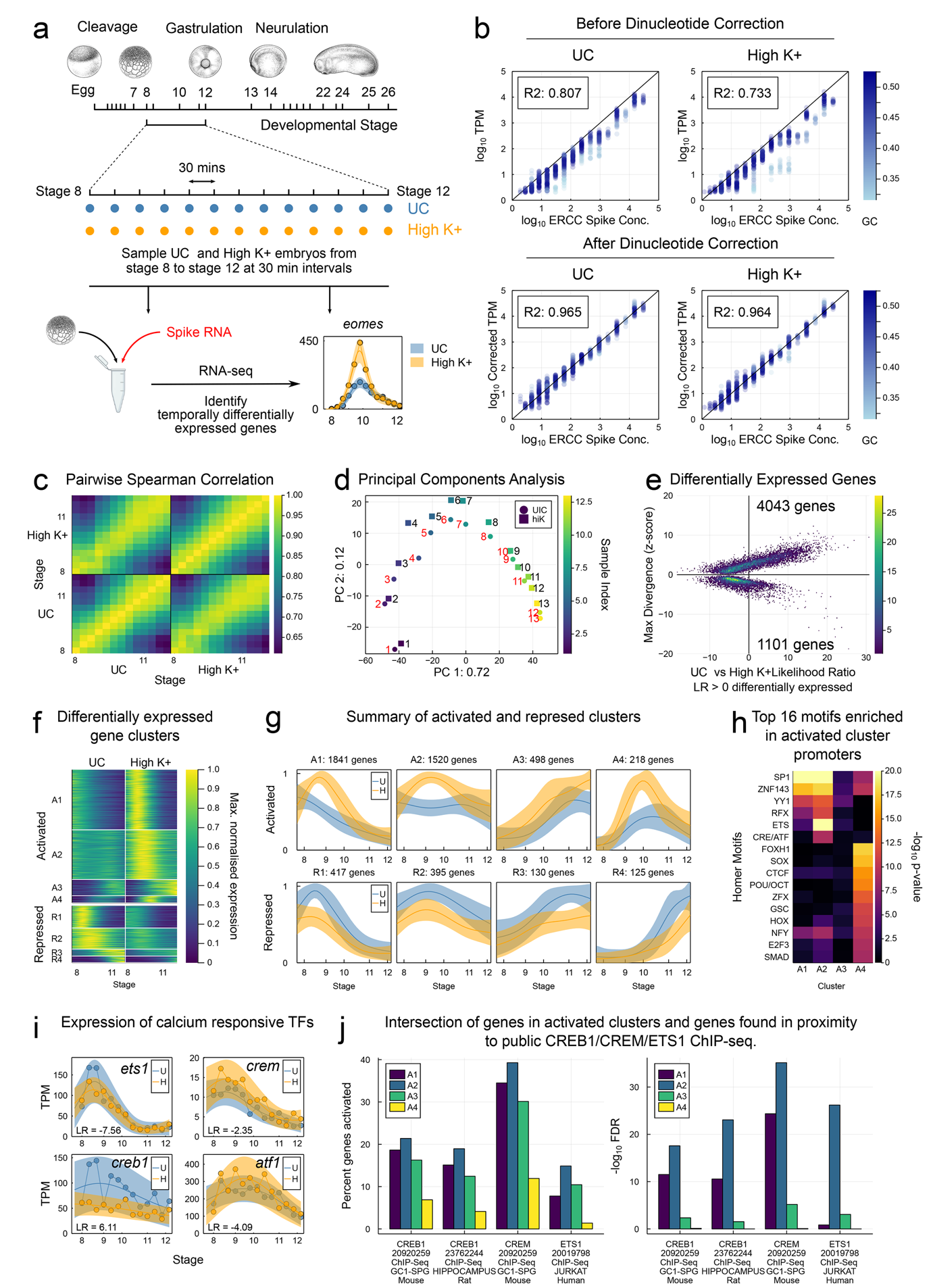


**Supplementary Figure 7. High Temporal Resolution RNAseq**

**a**) Schematic of RNAseq collection from stage 8 to stage 12. 10 embryos were collected every 30 mins from a clutch of synchronized embryos after IVF. *Xenopus* illustrations © Natalya Zahn (2022) from Xenbase (www.xenbase.org RRID:SCR_003280).

**b**) Quantification of ERCC spike-in transcripts before (top) and after (bottom) dinucleotide correction to account for GC bias for UC (=Untreated) Control and High K^+^ embryos. Note that true spike-in concentration is on the horizontal axis, and spikes with low GC content are underrepresented in sequencing. Corrected quantifications are used in all subsequent panels.

**c)** Pairwise Spearman correlation between all UC and High K+ samples after filtering for genes with sufficient temporal expression

**d**) Principal components analysis of log(TPM + 1) transformed expression for UC and HK+=High K^+^ samples. Sample index 1-13 labelled.

**e**) Visualisation of total differentially expressed genes and their magnitude as 2D histogram, colour indicating the frequency of genes in each bin. Horizontal axis gives log-likelihood-ratio, with LR > 0 defined as differentially expressed. Vertical axis gives maximal divergence z-score of UIC and High K^+^ trajectories in transformed GP space, averaging signal and noise variance for UC and High K^+^.

**f-g**) K-means clustering of activated and repressed genes as (f) heatmap and (g) cluster average. Shaded region in (f) gives +/- 1 SD for each cluster as in Main Fig. 6b.

**h**) Top 16 motif enrichments promoters (500 bp upstream of TSS) of activated clusters A1, A2, A3, A4. Heatmap gives –log10 Hypergeometric right tail p-value sorted by maximal enrichments such that motifs enriched in A1/A2 are at the top and those in A4 are at the bottom.

**i**) Expression of calcium responsive TFs, *ets1*, *crem*, *creb1*, *atf1*, demonstrating that these genes are not transcriptionally activated over our time course. Log-likelihood ratio given with LR > 0 differentially expressed. Central line and shaded region are transformed Gaussian process median and 95% CI.

**j)** Enrichments of public ChIP-seq peaks for CREB1, CREM and ETS in tissues and cell lines given in Human, Mouse and Rat, in proximity to genes in activated clusters A1, A2, A3, A4. Enrichments calculated with Enrichr and ChEA_2016 gene set^7^ which integrates data from a broad range of publicly available ChIP assays. ChEA_2016 term names given on horizontal axis describing target, Pubmed ID, cell/tissue and organism. Left panel gives size of intersection as percent of total genes in each cluster, right panel gives –log_10_ FDR for Fisher’s Exact test for overrepresentation in each cluster.


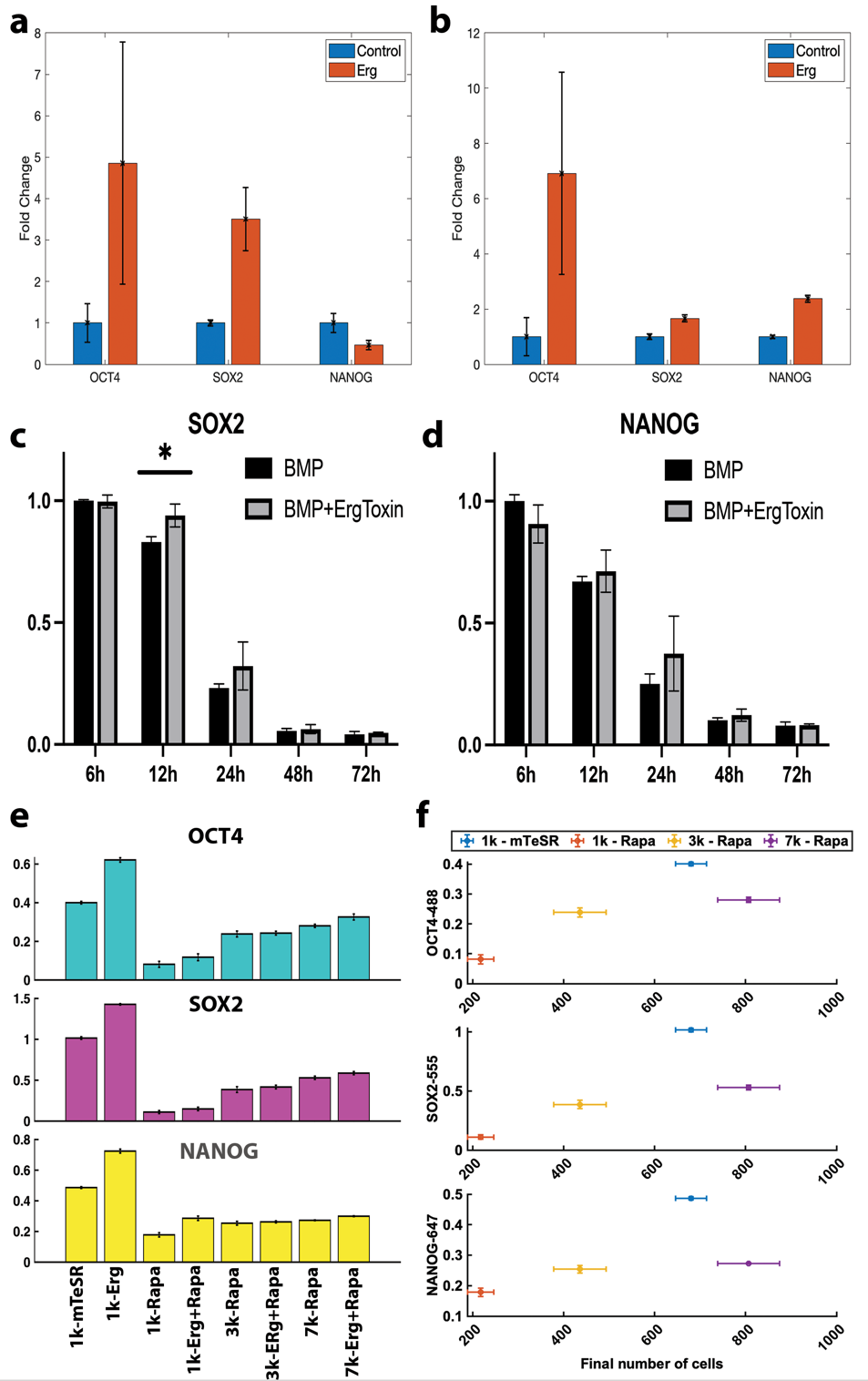


**Supplementary Figure 8. Blocking KCNH channels slows differentiation of hESCs while rapamycin treatment induces differentiation.**

**a and b)** Cells were grown with or without ErgToxin reagent (25 nM) for 2 (a) or 5 days (b) and then the indicated pluripotency markers were measured by qRT-PCR; mean fold-change (±SEM) over control hESCs is shown; data from 3 independent experiments.

**c and d)** Quantification of immunofluorescence against SOX2 (c) and NANOG (d) following treatment with 50 ng/ml BMP4 with or without Ergtoxin addition; presentation is in AU=arbitrary units; mean ±SEM from 3 independent experiments; *=p≤0.05; two-tailed unpaired student’s t-test.

**e)** Expression of pluripotency markers OCT4, SOX2 and NANOG in hESCs (relative immunofluorescence intensity, arbitrary units) upon treatment with rapamycin alone, or rapamycin and ErgToxin together, after seeding at different densities. 1000, 3000, or 7000 cells were seeded into an 18 well slide (Ibidi) and treated with rapamycin (100 nM) for 5 days. As a control, 1000 cells were seeded and grown without rapamycin treatment. Mean ±SEM from 3 independent experiments.

**f)** Pluripotency marker expression data from (e) plotted as a function of final cell density (relative immunofluorescence intensity, arbitrary units). Numbers indicated for final density are the average number of cells in one image, whose dimensions correspond to 0.636mmx0.636mm. Pluripotency markers are reduced in the rapamycin treated cells regardless of whether the final density is higher or lower than in the control condition. Mean ±SEM from 3 independent experiments.

Source data are provided as a Source Data file.

**Supplementary Table 1**

| **Protein** | **Species** | **Dilution** | **Catalog No.** | **Vendor** |
| --- | --- | --- | --- | --- |
| Oct4 | Mouse | 1:400 | 611203 | BD Biosciences |
| Sox2 | Rabbit | 1:200 | 5024S | CellSignalingTech |
| Nanog | Goat | 1:200 | AF1997 | R&D Systems |
| Nanog | Mouse | 1:400 | 560482 | BD Biosciences |
| Cdx2 | Mouse | 1:100 | MU392A-5UC | Biogenex |
| Eomes | Rabbit | 1:400 | Ab23345 | Abcam |
| Brachyury | Goat | 1:300 | AF2085 | R&D Systems |
| Isl1 | Mouse | 1:50 | 39.4D-5 | DSHB |

**Supplementary Table 1. Antibodies and dilutions used in this study.**

SUPPLEMENTARY REFERENCES

1 Dobin, A. *et al.* STAR: ultrafast universal RNA-seq aligner. *Bioinformatics* **29**, 15-21, doi:10.1093/bioinformatics/bts635 (2013).

2 Li, B. & Dewey, C. N. RSEM: accurate transcript quantification from RNA-Seq data with or without a reference genome. *BMC Bioinformatics* **12**, 323, doi:10.1186/1471-2105-12-323 (2011).

3 Owens, N. D. *et al.* Measuring Absolute RNA Copy Numbers at High Temporal Resolution Reveals Transcriptome Kinetics in Development. *Cell reports* **14**, 632-647, doi:10.1016/j.celrep.2015.12.050 (2016).

4 Kuleshov, M. V. *et al.* Enrichr: a comprehensive gene set enrichment analysis web server 2016 update. *Nucleic Acids Res* **44**, W90-97, doi:10.1093/nar/gkw377 (2016).

5 Heinz, S. *et al.* Simple combinations of lineage-determining transcription factors prime cis-regulatory elements required for macrophage and B cell identities. *Mol Cell* **38**, 576-589, doi:10.1016/j.molcel.2010.05.004 (2010).

6 Palmer, J. F. & Slack, C. Some bio-electric parameters of early Xenopus embryos. *J Embryol Exp Morphol* **24**, 535-553 (1970).

7 Lachmann, A. *et al.* ChEA: transcription factor regulation inferred from integrating genome-wide ChIP-X experiments. *Bioinformatics* **26**, 2438-2444, doi:10.1093/bioinformatics/btq466 (2010).
