# Supplementary material for: Membrane potential drives the exit from pluripotency and cell fate commitment via calcium and mTOR
Source: Nat Commun. 2022 Nov 5;13:6681. doi: 10.1038/s41467-022-34363-w (PMC9637099; doi:10.1038/s41467-022-34363-w)
Supplement: Supplementary file 3 — Description of Additional Supplementary Files [file 41467_2022_34363_MOESM3_ESM.pdf]

Description of Additional Supplementary Files

File Name: Supplementary Data 1
Description: Gene set enrichments for differentially expressed genes activated and repressed clusters.

File Name: Supplementary Movie 1
Description: Time lapse recording of GCaMP6 fluorescence in the animal pole of control MO stage 10 embryo. Duration= 20s (1 frame per second).

File Name: Supplementary Movie 2
Description: Time lapse recording of GCaMP6 fluorescence in the animal pole of kcnh6 MO stage 10 embryo. Duration= 20s (1 frame per second).
