# Supplementary material for: Membrane potential drives the exit from pluripotency and cell fate commitment via calcium and mTOR
Source: Nat Commun. 2022 Nov 5;13:6681. doi: 10.1038/s41467-022-34363-w (PMC9637099; doi:10.1038/s41467-022-34363-w)
Supplement: Supplementary file 7 — Reporting Summary [file 41467_2022_34363_MOESM7_ESM.pdf]

Corresponding author(s): Khokha, Mustafa K

Last updated by author(s): Jun 1, 2022

# Reporting Summary

Nature Portfolio wishes to improve the reproducibility of the work that we publish. This form provides structure for consistency and transparency in reporting. For further information on Nature Portfolio policies, see our Editorial Policies and the Editorial Policy Checklist.

## Statistics

For all statistical analyses, confirm that the following items are present in the figure legend, table legend, main text, or Methods section.

| n/a | Confirmed | |
|---|---|---|
| ☐ | ☒ | The exact sample size (*n*) for each experimental group/condition, given as a discrete number and unit of measurement |
| ☐ | ☒ | A statement on whether measurements were taken from distinct samples or whether the same sample was measured repeatedly |
| ☐ | ☒ | The statistical test(s) used AND whether they are one- or two-sided<br>*Only common tests should be described solely by name; describe more complex techniques in the Methods section.* |
| ☐ | ☒ | A description of all covariates tested |
| ☐ | ☒ | A description of any assumptions or corrections, such as tests of normality and adjustment for multiple comparisons |
| ☐ | ☒ | A full description of the statistical parameters including central tendency (e.g. means) or other basic estimates (e.g. regression coefficient) AND variation (e.g. standard deviation) or associated estimates of uncertainty (e.g. confidence intervals) |
| ☐ | ☒ | For null hypothesis testing, the test statistic (e.g. *F*, *t*, *r*) with confidence intervals, effect sizes, degrees of freedom and *P* value noted<br>*Give P values as exact values whenever suitable.* |
| ☐ | ☒ | For Bayesian analysis, information on the choice of priors and Markov chain Monte Carlo settings |
| ☐ | ☒ | For hierarchical and complex designs, identification of the appropriate level for tests and full reporting of outcomes |
| ☐ | ☒ | Estimates of effect sizes (e.g. Cohen's *d*, Pearson's *r*), indicating how they were calculated |

*Our web collection on statistics for biologists contains articles on many of the points above.*

## Software and code

Policy information about availability of computer code

| | |
|---|---|
| Data collection | no software |
| Data analysis | All source data and code required to analyse RNA-seq time series and generate figures is available at https://github.com/owensnick/KCNH6GenomicsFigures.jl. Source code for quantitating protein intensities in human ES cells is available at https://github.com/warmflashlab/Sempou2022_Code. |

For manuscripts utilizing custom algorithms or software that are central to the research but not yet described in published literature, software must be made available to editors and reviewers. We strongly encourage code deposition in a community repository (e.g. GitHub). See the Nature Portfolio guidelines for submitting code & software for further information.

## Data

Policy information about availability of data

All manuscripts must include a data availability statement. This statement should provide the following information, where applicable:

- Accession codes, unique identifiers, or web links for publicly available datasets
- A description of any restrictions on data availability
- For clinical datasets or third party data, please ensure that the statement adheres to our policy

RNA-seq time series in uninjected-control (UIC) and high K+ are available at the Gene Expression Omnibus, under accession GSE186670.

## Human research participants

Policy information about studies involving human research participants and Sex and Gender in Research.

| | |
|---|---|
| Reporting on sex and gender | N/A |
| Population characteristics | N/A |
| Recruitment | N/A |
| Ethics oversight | N/A |

Note that full information on the approval of the study protocol must also be provided in the manuscript.

# Field-specific reporting

Please select the one below that is the best fit for your research. If you are not sure, read the appropriate sections before making your selection.

☒ Life sciences ☐ Behavioural & social sciences ☐ Ecological, evolutionary & environmental sciences

For a reference copy of the document with all sections, see nature.com/documents/nr-reporting-summary-flat.pdf

# Life sciences study design

All studies must disclose on these points even when the disclosure is negative.

| | |
|---|---|
| Sample size | No statistical methods were used to predetermine sample size. We performed experiments and then calculated statistical significance using testing appropriate for the comparisons made. In all cases, at least three biological replicates were done, and all sample sizes are reported in the figures. Generating large numbers of embryos in Xenopus is straightforward. |
| Data exclusions | no data was excluded |
| Replication | All experiments include three independent biological replicates except for RNAseq where multiple time points were collected from a single biological replicate. There were no failed attempts at replication that were excluded. |
| Randomization | For all Xenopus experiments, embryos were selected for control or manipulated groups randomly. For all human stem cell experiements, cells were distributed across manipulated or control conditions also randomly. |
| Blinding | Embryos and cells were scored blindly and conditions were only revealed after the scoring was complete. |

# Reporting for specific materials, systems and methods

We require information from authors about some types of materials, experimental systems and methods used in many studies. Here, indicate whether each material, system or method listed is relevant to your study. If you are not sure if a list item applies to your research, read the appropriate section before selecting a response.

## Materials & experimental systems

| n/a | Involved in the study | |
|---|---|---|
| ☐ | ☒ | Antibodies |
| ☐ | ☒ | Eukaryotic cell lines |
| ☒ | ☐ | Palaeontology and archaeology |
| ☐ | ☒ | Animals and other organisms |
| ☒ | ☐ | Clinical data |
| ☒ | ☐ | Dual use research of concern |

## Methods

| n/a | Involved in the study | |
|---|---|---|
| ☒ | ☐ | ChIP-seq |
| ☒ | ☐ | Flow cytometry |
| ☒ | ☐ | MRI-based neuroimaging |

## Antibodies

Antibodies used

Protein Species Dilution Catalog No. Vendor
Oct4 Mouse 1:400 611203 BD Biosciences
Sox2 Rabbit 1:200 5024S CellSignalingTech
Nanog Goat 1:200 AF1997 R&D Systems

| | |
|---|---|
| | Nanog Mouse 1:400 560482 BD Biosciences<br>Cdx2 Mouse 1:100 MU392A-5UC Biogenex<br>Eomes Rabbit 1:400 Ab23345 Abcam<br>Brachyury Goat 1:300 AF2085 R&D Systems<br>Isl1 Mouse 1:50 39.4D-5 DSHB<br>MyoD Rabbit 1:100 C143580-100 LsBio<br>acetylated tubulin mouse 1:2000 T-6793 Sigma |
| Validation | These are standard antibodies validated in a host of previous publications. |

## Eukaryotic cell lines

Policy information about cell lines and Sex and Gender in Research

| | |
|---|---|
| Cell line source(s) | The cell lines used were ESI017 (ESIBIO) and H9 (Thomson et al Science 282:1145 (1998)) |
| Authentication | Cells were routinely tested for expression of all three pluripotency markers (Oct4, Sox2, Nanog) to ensure maintenance of pluripotency. |
| Mycoplasma contamination | tested for mycoplasma and found negatoive. |
| Commonly misidentified lines<br>(See ICLAC register) | none |

## Animals and other research organisms

Policy information about studies involving animals; ARRIVE guidelines recommended for reporting animal research, and Sex and Gender in Research

| | |
|---|---|
| Laboratory animals | Xenopus tropicalis, nigerian, adult, females and males, 2-5 years old. |
| Wild animals | none |
| Reporting on sex | all experiments were concluded before sex could be determined. Sex is genetically defined in Xenopus with 50:50 percent ratios. |
| Field-collected samples | none |
| Ethics oversight | Yale IRB |

Note that full information on the approval of the study protocol must also be provided in the manuscript.
